# High Platelet Reactivity in Patients with Acute Coronary Syndromes Undergoing Percutaneous Coronary Intervention: Randomised Controlled Trial Comparing Prasugrel and Clopidogrel

**DOI:** 10.1371/journal.pone.0135037

**Published:** 2015-08-28

**Authors:** Tobias Geisler, Jean Booth, Elli Tavlaki, Athanasios Karathanos, Karin Müller, Michal Droppa, Meinrad Gawaz, Monica Yanez-Lopez, Simon J. Davidson, Rod H. Stables, Winston Banya, Azfar Zaman, Marcus Flather, Miles Dalby

**Affiliations:** 1 Klinikum der Eberhard-Karls-Universität Tübingen, Abteilung für Kardiologie und Kreislauferkrankungen, Tübingen, Germany; 2 Clinical Trials & Evaluation Unit, Royal Brompton Hospital, London, United Kingdom; 3 Dept. Haematology, Royal Brompton&Harefield NHS Foundation Trust, London, United Kingdom; 4 Liverpool Heart and Chest Hospital, Liverpool, United Kingdom; 5 Freeman Hospital and Institute of Cellular Medicine, Newcastle University, Newcastle upon Tyne, United Kingdom; 6 Norfolk and Norwich University Hospitals NHS Foundation Trust and Norwich Medical School, University of East Anglia, Norfolk, United Kingdom; 7 Harefield Hospital, Royal Brompton & Harefield NHS Foundation Trust, London, United Kingdom; Maastricht University Medical Center, NETHERLANDS

## Abstract

**Background:**

Prasugrel is more effective than clopidogrel in reducing platelet aggregation in acute coronary syndromes. Data available on prasugrel reloading in clopidogrel treated patients with high residual platelet reactivity (HRPR) i.e. poor responders, is limited.

**Objectives:**

To determine the effects of prasugrel loading on platelet function in patients on clopidogrel and high platelet reactivity undergoing percutaneous coronary intervention for acute coronary syndrome (ACS).

**Patients:**

Patients with ACS on clopidogrel who were scheduled for PCI found to have a platelet reactivity ≥40 AUC with the Multiplate Analyzer, i.e. “poor responders” were randomised to prasugrel (60 mg loading and 10 mg maintenance dose) or clopidogrel (600 mg reloading and 150 mg maintenance dose). The primary outcome measure was proportion of patients with platelet reactivity <40 AUC 4 hours after loading with study medication, and also at one hour (secondary outcome). 44 patients were enrolled and the study was terminated early as clopidogrel use decreased sharply due to introduction of newer P2Y12 inhibitors.

**Results:**

At 4 hours after study medication 100% of patients treated with prasugrel compared to 91% of those treated with clopidogrel had platelet reactivity <40 AUC (p = 0.49), while at 1 hour the proportions were 95% and 64% respectively (p = 0.02). Mean platelet reactivity at 4 and 1 hours after study medication in prasugrel and clopidogrel groups respectively were 12 versus 22 (p = 0.005) and 19 versus 34 (p = 0.01) respectively.

**Conclusions:**

Routine platelet function testing identifies patients with high residual platelet reactivity (“poor responders”) on clopidogrel. A strategy of prasugrel rather than clopidogrel reloading results in earlier and more sustained suppression of platelet reactivity. Future trials need to identify if this translates into clinical benefit.

**Trial Registration:**

ClinicalTrials.gov NCT01339026

## Introduction

Dual antiplatelet therapy (DAPT) consisting of aspirin and an adenosine diphosphate (ADP) receptor inhibitor has been shown to reduce the risk of subsequent vascular events including myocardial infarction (MI) and stent thrombosis in patients with acute coronary syndromes (ACS).[1] Some of the benefits of DAPT for ACS patients undergoing percutaneous coronary intervention (PCI) appear to be related to pre-treatment with a loading dose of the ADP-receptor blocker clopidogrel.[2] In a substantial proportion of patients clopidogrel is associated with poor antiplatelet response [3] probably due to restriction of bioavailability and inter patient variation due to phenotype or genotype. Although prasugrel and ticagrelor exhibit faster onset of action and reach better clinical outcomes with enhanced platelet inhibition prior to PCI, the majority of patients continue to receive clopidogrel as reported in the European registry APTOR [4]. Clopidogrel has shortcomings with slow onset of action and high variability of platelet inhibition caused by genetically defined poor metabolism leading to more than half of patients exhibiting continued high platelet reactivity at the time of PCI, despite a timely administered high dose loading.[5] High platelet reactivity is associated with a higher risk of thrombotic events.[6] Accordingly we established the APACS HPR (Additional Platelet inhibition in Acute Coronary Syndromes with High Platelet Reactivity) trial to measure the effects on platelet function of an additional loading dose of prasugrel, or clopidogrel, in patients with ACS scheduled for PCI who had been started on clopidogrel but had high platelet reactivity (“poor responders”).

## Methods

### Study Design

APACS was a randomised, open label study carried out in 1 centre in Germany and 4 centres in the UK comparing prasugrel with clopidogrel reloading in ACS patients pre-treated with clopidogrel who had high residual platelet reactivity (“poor responders”). PCI had to be planned to take place as early as possible and no later than 72 hours from admission.

**Fig 1:**
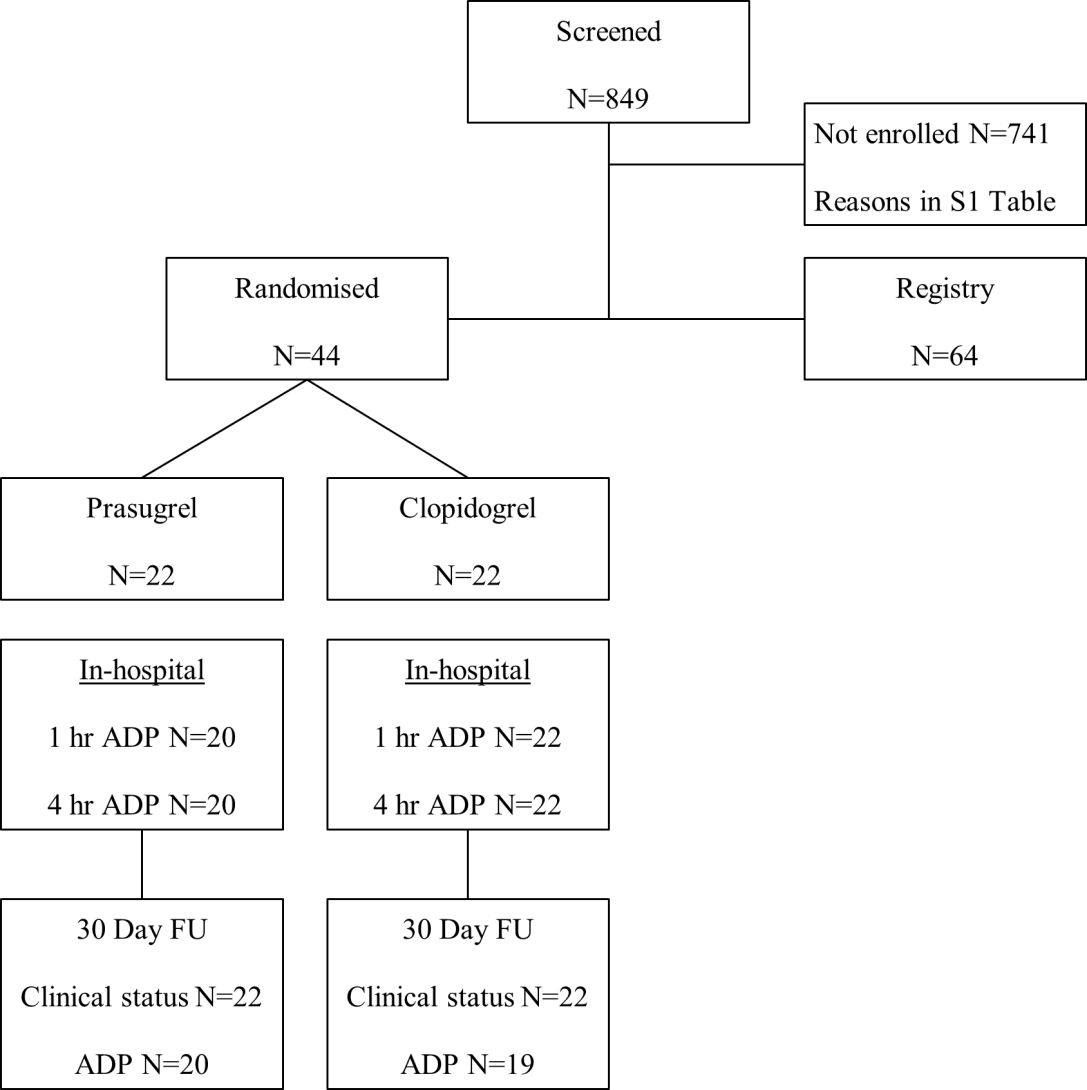
Consort flow diagram of the trial.

**Fig 2:**
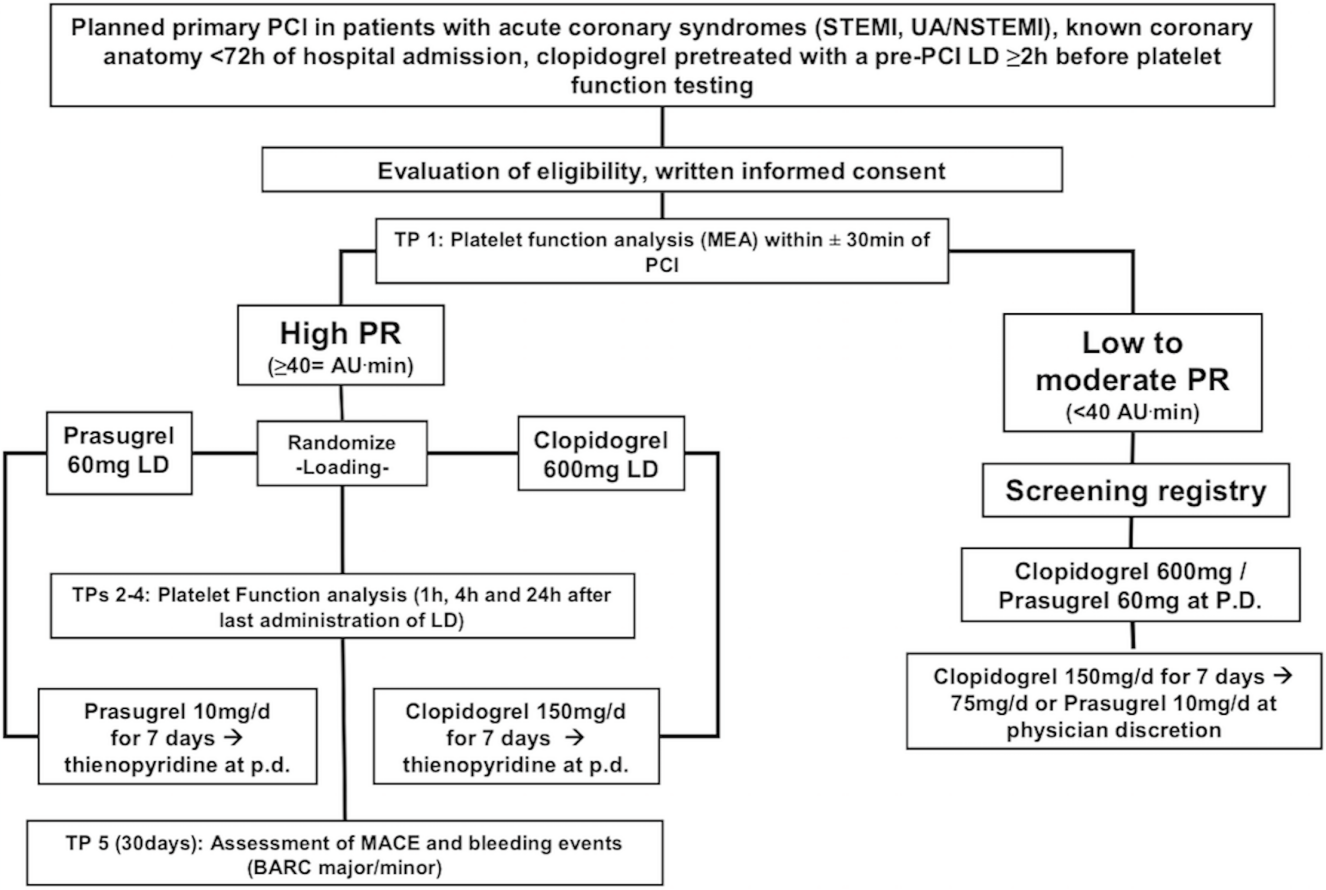
Diagram of inclusion and randomisation process (Abbreviations: BARC: Bleeding Academic Research Consortium; LD: Loading Dose, MACE: major adverse cardiovascular events, MEA: Multiple Electrode Aggregometry; PR: Platelet reactivity, TP: Time point, P.D.: Physician’s Discretion).

Patients with prior clopidogrel loading within 24h before planned PCI or receiving chronic treatment with clopidogrel (e.g. for >24 hours having received at least one previous 600 mg loading dose with subsequent 75 mg maintenance dose, or ≥7 days of maintenance therapy) who had high platelet reactivity ≥ 40 AUC were randomised to prasugrel (60 mg loading and 10 mg maintenance dose) or a high dose clopidogrel regimen (600 mg reloading followed by 150 mg maintenance dose). There are no large clinical endpoint studies evaluating cut-off values for platelet reactivity measured with Multiplate Analyzer in the early phase e.g. 4h after loading dose in ACS patients to predict early periprocedural events. 40 AUC was a prespecified arbitrary cut-off value based on previous observations, showing that a similar degree of platelet reactivity can be achieved at ~4h after loading with newer platelet inhibitors prasugrel and ticagrelor. [7] APACS was an open-label trial. The laboratory assistant performing the platelet function analysis was blinded to the allocation arms. Flow diagram of the study is shown in Fig 1, diagram of randomisation process in Fig 2.

Screened patients identified with low platelet reactivity indicating a good response to clopidogrel were entered into a registry. The trial was registered at clinicaltrial.gov (NCT01339026). The APACS study was conducted in compliance with the principles of the Declaration of Helsinki and the International Conference on Harmonization consolidated guidelines and was approved by the NHS Research Ethics Committee and the ethical review committees of Tübingen University. Patients gave written informed consent prior to study participation.

### Patients

Inclusion criteria were: ACS, age: 18–75 years, intention to perform PCI <72 hours from admission, prior or chronic treatment with clopidogrel (defined as prior clopidogrel loading within 24h before planned PCI or chronic >24 hours and at least 7 days of maintenance dose treatment with clopidogrel), high platelet reactivity as defined by ADP induced platelet activation of ≥ 40 AUC by Multiplate analyser with timing of platelet function assay at least 2 hours after pre-PCI loading dose, provision of informed consent by participating patient. Inclusion criteria for the registry were the same as above except for a platelet reactivity of <40 AUC.

Exclusion Criteria were body weight <60 kg, pre-treatment with prasugrel within 7 days of randomisation, history of stroke or transient ischemic attack, patients with increased bleeding risk e.g. recent major trauma or surgery, gastrointestinal bleeding or active peptic ulceration, platelet count < 100 x10^9^/L at the time of screening, International Normalized Ratio (INR)> 1.5 at the time of screening, Hb<10g/dL, intracranial neoplasm, arteriovenous malformation or aneurysm, severe hepatic impairment (Child Pugh class C) or intention to use the following medications: oral anticoagulation, other antiplatelet therapy (including GPIIb/IIIa inhibitors) besides aspirin, non-steroidal anti-inflammatory drugs (NSAIDs) or cyclooxygenase-2 (COX-2) inhibitors, female patients who are pregnant, planning pregnancy, or who are breastfeeding, known allergy, hypersensitivity or other contraindications to prasugrel or clopidogrel or hemodynamically unstable patients in whom study related procedures might cause unnecessary delays to urgent revascularization.

### Impedance platelet aggregometry

The Multiplate analyzer, a whole blood platelet function assay, was used to study the platelet aggregation level. A 600μL blood sample acquired in hirudinized tubes (Sarstedt) was obtained to perform ADP and TRAP tests in each patient. The blood samples were taken before the loading dose, 1h, 4 hours and 24h after the loading dose. The area under the aggregation curve (AUC) was used as a measure of the overall platelet aggregation. Tests were performed 30 minutes to 3 hours after taking blood.

### Randomisation and treatment schedule

Patients were randomised to either open label clopidogrel (600 mg additional loading dose, followed by 150 mg daily maintenance dose for 7 days, followed by 75 mg daily for up to 12 months) or prasugrel (60 mg additional loading dose, followed by 10 mg daily). The randomisation algorithm is presented in Fig 2. Randomisation blocks were generated by the responsible statistician (Winston Banya) and sent to Sealed Envelope for the web based randomisation database. The investigator or his/her designee accessed the randomisation service and the randomisation allocation was released after eligibility criteria were confirmed and informed consent had been obtained. Each randomised patient was assigned a unique identifying number. The web based database system sent automatic email confirmation of randomisation to authorised personnel.

### Follow Up

Randomised patients were followed up to 30 days for clinical status, study medication, blood samples for adenosine diphosphate ADP) and thrombin receptor activating peptide (TRAP) test and serious adverse events (SAEs). Registry patients were followed up to 30 days for survival. Major Adverse Cardiac Events (MACE) were defined as death, stroke, myocardial infarction and repeat revascularization. Bleeding events were defined according to the bleeding academic research consortium criteria (BARC) criteria as previously reported.[8]

Primary endpoint was the proportion of patients with improved platelet response i.e. platelet reactivity under the cut-off value of 40 AUC in the prasugrel reloading arm compared to the clopidogrel reloading arm at 4 hours after randomisation in patients with initial high platelet reactivity.

The secondary endpoints were ADP induced platelet reactivity at 1 and 24 hours, TRAP induced platelet reactivity at 1, 4 and 24 hours, and MACE including death, stroke, myocardial infarction, repeat revascularization and major bleedings defined according to BARC.

### Statistical analysis

For an absolute difference of 20% in conversion of patients with initial high platelet reactivity to a platelet reactivity under the cut-off value of <40 AUC (i.e. reduction of prevalence from 30% to at least 10%) after reloading with prasugrel compared to clopidogrel the estimated minimum sample size was 62 patients per arm (80% power at a two-sided alpha value of 5%) to detect this difference. Baseline variables were compared with the use of chi-square tests for categorical variables; t-tests were used for normally distributed continuous variables and the Wilcoxon rank-sum for variables with non-Gaussian distributions. Comparison of the primary endpoint and secondary endpoints was carried out using a chi squared or Fishers exact test. The confidence intervals were two-sided with a 95% confidence level, and all hypothesis tests were two-sided carried out at a significance level of 0.05.

## Results

The study started enrolment in February 2012. During the course of the trial clinicians had started to adopt clinical guidelines recommending prasugrel or ticagrelor over clopidogrel for ACS patients.[9,10] which decreased the use of clopidogrel. A decision was therefore made by the APACS Investigators group to terminate recruitment on the 31st July 2013 well before the predicted recruiting time, so that only 44 patients could be recruited.

**Fig 3:**
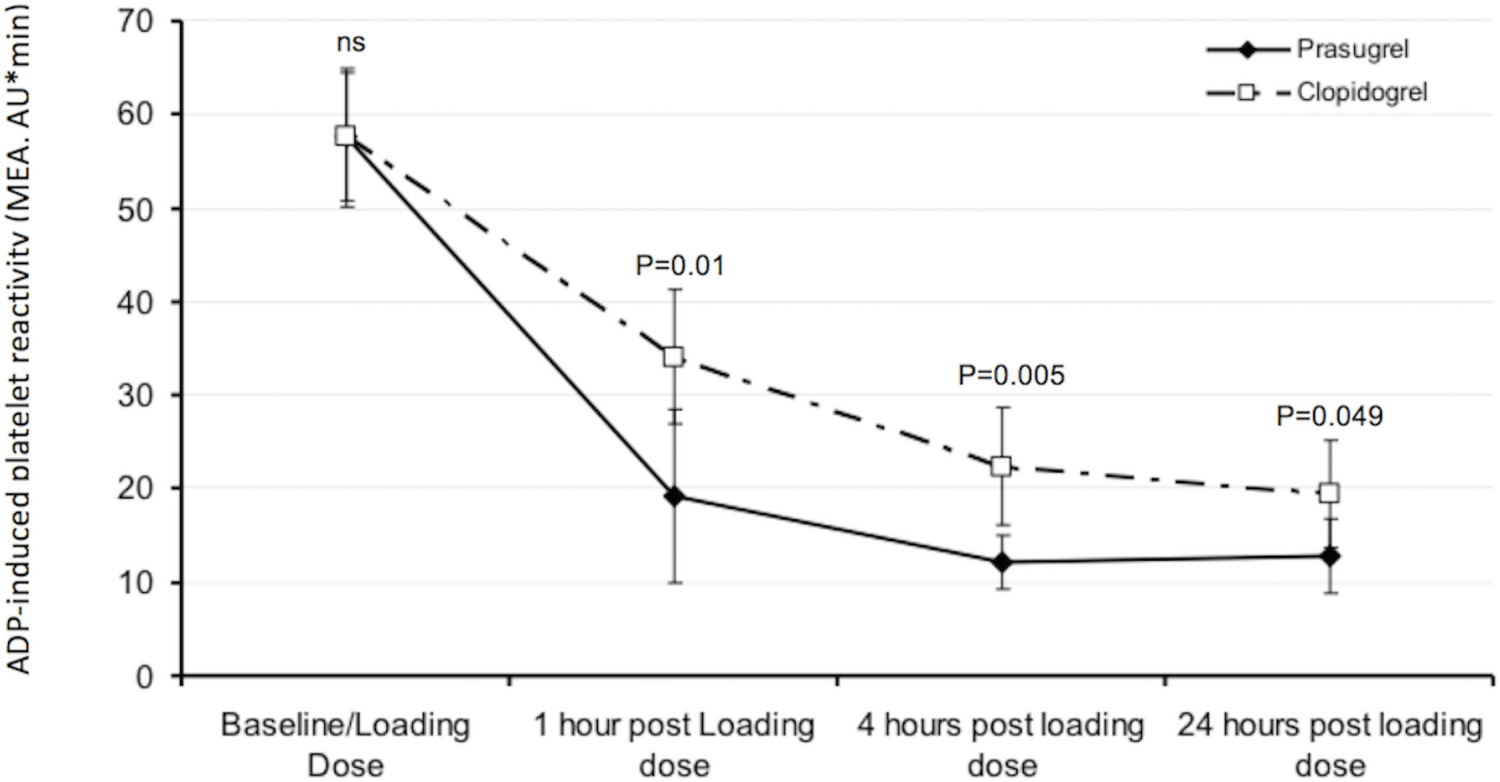
Pharmacodynamic profile showing ADP induced platelet aggregation over time according to treatment arms.

Of the ACS cohort 40.7% was found to have HRPR at the time of PCI despite pre-treatment with clopidogrel. 44 patients fulfilled all eligibility criteria, consented to study participation and were enrolled. Another 64 patients fulfilled eligibility criteria but had normal platelet reactivity (i.e. <40 AUC) and entered the registry (Fig 1). The baseline characteristics of randomised and screened patients are shown in Table 1. Patients with high platelet reactivity versus normal platelet reactivity were more often hypercholesterolemic or diabetic. The mean age of the randomised patients was 59.7 years and 86% (n = 38) were male. Severe angina (CCS class III or IV) was present in 39% (n = 17) of patients. 41% (n = 18) had diabetes, 34% (n = 15) had a prior myocardial infraction (MI) and 51% (n = 22) had a prior PCI (Table 1). Baseline characteristics of demographic and clinical parameters were well balanced between randomisation arms (Table 2). Baseline mean platelet reactivity was 57.6 AUC in both groups as shown in Table 3. The proportion of patients who changed from ADP ≥ 40 AUC to < 40 AUC are shown in Table 3 and the change over time is shown in Fig 3. Mean values for platelet aggregation over time are shown in Fig 3 confirming that prasugrel was more effective in reducing platelet aggregation. For the primary outcome analysis at 4 hours post loading with the study drug there was no significant difference between the groups, p = 0.49. Nevertheless, at 1 hour post loading with prasugrel 95% of patients had <40 AUC compared to 64% in the clopidogrel group (p = 0.022). The change in platelet reactivity (from clopidogrel to prasugrel) at 1 hour was 14.85 AUC (95% CI 3.64, 26.05, p = 0.01) and at 4 hours was 10.26 AUC (95% CI 3.29, 17.24, p = 0.005).

**Table 1:** Baseline characteristics in the randomised patients versus screened patients. Abbreviations: LBBB: left bundle branch block, RBBB: right bundle branch block, BMI: body mass index.

Variable	Randomised (n = 44)	Registry (n = 64)	P
Age–mean (SD)	59.6 (9.2)	62.3 (9.6)	0.15
Gender—Male	38 (86.4)	46 (71.9)	0.08
BMI–mean (SD)	29.2 (5.4)	28.4 (3.9)	0.43
Ethnicity: White European South Asian/ other	: 40/44 (91): 4/44 (9)	: 63/63 (100): 0/63 (0)	
systolic blood pressure: –mean (SD)	138 (24)	134 (20)	0.32
diastolic blood pressure: –bmean (SD)	76 (13)	75 (12)	0.81
Heart rate–mean (SD)	71 (19)	70 (11)	0.73
Heart Rhythm: Sinus rhythm: Atrial fibrillation: Paced: Other	: 39 (88.64): 2 (4.55): 1 (2.27): 2 (4.55)	: 61/63 (96.83): 1/63 (1.59): 0: 1/63 (1.59)	0.25
ECG Changes: No change: T Wave Inversion: ST Depression: ST Elevation: LBBB:: RBBB: Other changes	: 13 (29.55): 18 (36.36): 1 (2.27): 3 (6.82): 2 (4.55): 1 (2.27): 8 (18.18)	: 26/63 (41.27): 18/63 (28.57): 9/63 (14.29): 7/63 (11.11): 2/63 (3.17): 1/63 (1.59): 0	
Hypertension	30 (68.2)	35/62 (56.45)	0.22
Hypercholesterolemia	27 (61.4)	26/63 (41.27)	0.04
Diabetes	18 (40.9)	14/63 (22.22)	0.038
Prior MI	15 (34.1)	14 (21.88)	0.16
Prior PCI	22/43 (51.2)	18 (28.13)	0.016
Smoking history: None:: Ex: Current	:: 7/28 (25.0): 14/28 (50.0): 7/28 (25.0)	: 15/43 (34.88): 14/43 (32.56): 14/43 (32.56)	0.34
Loading Platelet reactivity: –mean (SD)	57.6 (15.7)	21.2 (10.6)	< 0.0001
Type of admission Ambulance Elective: Transfer from other hospital: Other	:: 31 (70.45): 7 (15.91): 6 (13.64): 0	:: 33 (51.56): 4 (6.25): 25 (39.06): 2 (3.13)	0.007

**Table 2:** Baseline characteristics according to randomisation arms.

Variable	Prasugrel (n = 22)	Clopidogrel (n = 22)	p
Age: mean (SD)	60.9 (9.9)	58.5 (8.5)	0.39
Gender–Male	19/22 (86.4)	19/22 (86.4)	1.00
BMI: mean (SD)	29.0 (4.4)	29.3 (6.4)	0.87
systolic blood pressure: –mean (SD)	141 (28)	135 (19)	0.40
diastolic blood pressure: –mean (SD)	74 (14)	78 (12)	0.51
Heart rate–mean (SD)	72 (20)	71 (18)	0.78
CCS Class: 0:: I: II:: III: IVa	: 7/20 (35.0): 1/20 (5.0): 1/20 (5.0): 1/20 (5.0): 10/20 (50.0)	:: 5/16 (31.25): 3/16 (18.75): 2/18 (12.50): 1/16 (6.25): 5/16 (31.25)	0.58
NYHA Class: I: II: III	: 13/22 (59.09): 7/22 (31.82): 2/22 (9.09)	:: 8/16 (50.00): 5/16 (31.25): 3/16 (18.75)	0.73
Heart Rhythm: Sinus rhythm: Atrial fibrillation: Paced: Other	: 20/22 (90.90): 1/22 (4.55): 1/22 (4.55): 0/22	: 19/22 (86.36): 1/22 (4.55): 0/22: 2/22 (9.09)	0.74
ECG Changes: No change: T Wave Inversion: ST Depression: ST Elevation: LBBB: RBBB: Other changes	: 8/22 (36.36): 9/22 (40.91): 0: 0: 1/22 (4.55): 0: 4/22 (18.18)	:: 5/22 (22.73): 7/22 (31.82): 1/22 (4.55): 3/22 (13.64): 1/22 (4.55): 1/22 (4.55): 4/22 (18.18)	0.45
ACS type: STEMI: NSTEMI: Unstable Angina	: 0 (0): 8 (36.4): 14 (53.8)	:: 3 (13.6): 7 (31.8): 12 (54.5)	0.32
Hypertension	14/22 (63.6)	16/22 (72.7)	0.52
Hypercholesterolemia	12/22 (55.6)	15/22 (68.2)	0.35
Diabetes	11/22 (50.0)	7/22 (31.8)	0.22
Prior MI	7/22 (31.8)	8/22 (36.4)	0.75
Prior PCI	11/22 (50.0)	11/21 (52.4)	0.87
Hb (mg/dL)–mean (SD)	14.4 (1.4)	14.2 (1.4)	0.69
Platelet count x1000/microL: –mean (SD)	24.4 (6.7)	27.5 (5.5)	0.12
Creatinine (mg/dl): –mean (SD)	86.8 (29.8)	78.0 (19.1)	0.27
Beta Blockers	17/22 (77.3)	13/22 (59.1)	0.20
ACE Inhibitors	9/22 (40.9)	12/22 (54.6)	0.37
Aspirin:: In Ambulance: On Arrival: Before admission	: 7/20 (35.0): 9/20 (45.0): 4/20 (20.0)	:: 7/19 (36.84): 12/19 (63.16): 0	0.15
Statins	13/22 (59.09)	15/22 (68.18)	0.53

**Table 3:** Multiplate results. ADP-Test of randomised patients at different time points before and after randomisation. Number of patients with high and low platelet reactivity in every arm are presented according to time and the mean platelet reactivity in AUC for each arm are presented

Time point of Multiplate	Patients in the Prasugrel arm (n = 22)	Patients in the Clopidogrel arm (n = 22)	Total patients (n = 44)	p
*t*_*0*_: *Loading dose administration*				
≥ 40 AUC	20/21 (95.24)	21/22 (95.45)	41/43 (95.35)	: 1.00
< 40 AUC	1/21 (4.76)	1/22 (4.55)	2/43 (4.65)	
*t*_*1*_: *1 Hour Post Loading Dose (secondary outcome)*				
≥ 40 AUC	1/20 (5.0)	8/22 (36.36)	9/42 (21.43)	: 0.022
< 40 AUC	19/20 (95.0)	14/22 (63.64)	33/42 (78.57)	
*t*_*2*_: *4 Hour Post Loading Dose (primary outcome)*				
≥ 40 AUC	0/20	2/22 (9.09)	2/42 (4.76)	: 0.49
< 40 AUC	20/20 (100.0)	20/22 (90.91)	40/42 (95.24)	
**Comparison of mean platelet reactivity (AUC)**				
Loading dose	57.6 (50.2, 65.0)	57.6 (50.7, 64.5)	57.58 (52.76, 62.41)	1.00
1 h post loading dose	19.2 (9.9, 28.5)	34.0 (26.9, 41.2)	26.98 (20.98, 32.97)	0.010
4 h post loading dose	12.1 (9.2, 15.0)	22.4 (16.1, 28.7)	17.48 (13.68, 21.27)	0.005
24 h post loading dose	12.7 (8.8, 16.8)	19.5 (13.8, 25.2)	16.1 (12.5, 19.6)	0.049

With respect to TRAP induced platelet aggregation there was a significant difference at baseline between both arms. Patients randomised to prasugrel had lower TRAP induced platelet aggregation compared to patients allocated to clopidogrel reloading (mean 98.6 AUC 95%CI 89.8, 101.4 vs mean 108.1 AUC 95%CI 96.5, 119.7; p = 0.02). The reduced TRAP induced aggregation values in the prasugrel compared to the clopidogrel treated arm persisted over time mean 66.9 AUC (95%CI 58.3, 75.5) vs. mean 78.9 AUC (95%CI 69.4, 88.3) at 1 hour, mean 63.0 AUC (95%CI 55.6, 70.4) vs. mean 67.4 (95%CI 58.8, 76.0, p = 0.23) at 24 hours; (Table 4).

**Table 4:** TRAP-Test results at different time points before and after randomisation (Mean platelet reactivity (95% CI).

Time point of Multiplate	TRAP-test in the Prasugrel arm	TRAP-test in the Clopidogrel arm	Mean TRAP-test result	P
Loading dose	88.2 (75.5, 100.8)	108.1 (96.5, 119.7)	98.6 (89.8, 101.4)	0.02
1 h post loading dose	53.8 (40.7, 66.8)	78.9 (69.4, 88.3)	66.9 (58.3, 75.5)	0.002
4 h post loading dose	53.9 (43.1, 64.8)	65.4 (55.8, 75.0)	60.1 (53.0, 64.2)	0.11
24 h post loading dose	58.6 (46.0, 71.2)	67.4 (58.8, 76.0)	63.0 (55.6, 70.4)	0.23

Clinical events are shown in Table 5. There were no deaths. In the prasugrel group, there was 1 MI, and 1 repeat revascularization reported. In the clopidogrel group there were 1 stroke and 1 repeat revascularization. In the prasugrel group there were 1 BARC major bleed type 3b and 2 BARC minor bleeds type 1. In the clopidogrel group there was 1 major bleed (Cerebrovascular accident) and no minor bleeds were reported.

**Table 5:** Patients with MACE or Bleedings.

Events	Prasugrel (n = 22)	Clopidogrel (n = 22)
MACE		
Death	0	0
Myocardial Infarction	1	0
Cerebral Vascular Accident	0	1
Revascularization	1	1
Bleeds (BARC definition)		
Major	1	1
Minor	2	0

## Discussion

The primary endpoint of the proportion of patients with platelet reactivity under the cut-off value of 40 AUC after randomisation was not significantly different in the prasugrel reloading arm compared to the clopidogrel reloading arm at 4 hours although this analysis lacks statistical power due to early termination. In the secondary analysis, reloading with prasugrel provided a better antiplatelet response at one hour than reloading with clopidogrel. The findings can be explained by the greater antiplatelet efficacy and faster onset of prasugrel shown in mechanistic studies and better clinical outcomes as seen in TRITON-TIMI38. However a direct comparison of these two agents in clopidogrel “poor responders” in the setting of ACS and PCI has not been carried out.

Poor platelet inhibition in the early phase of ACS and PCI is associated with MACE including acute stent thrombosis.[11] In a recent meta-analysis of randomised controlled trials reducing high platelet reactivity in ACS was associated with a reduction of major ischemic complications.[12] Prasugrel achieves higher platelet inhibition compared to clopidogrel in pharmacodynamic analyses in healthy volunteers and stable CAD patients.[13,14] In TRITON-TIMI38 prasugrel was compared to clopidogrel 300mg in P2Y_12_ inhibitor naïve patients. To date there is no data from randomised trials comparing the peri-procedural effects of prasugrel versus high dose clopidogrel in thienopyridine pre-treated ACS patients which is a common clinical scenario. In fact, randomised studies investigating pharmacodynamic/pharmacokinetic profile suggest that even with the third generation P2Y_12_ inhibitors prasugrel and ticagrelor, the platelet inhibitory effect in real world ACS patients is insufficient at the time of the procedure.[15,16] This effect might be partly caused by the higher degree of platelet activation and aggregation in the setting of an acute coronary event and partly because of response variability due to genetic and clinical factors also seen with 3^rd^ generation P2Y_12_ receptor blockers. On the other hand, early and potent platelet inhibition by prasugrel loading in the upstream period has been associated with an excess of procedural related major bleedings without ischemic risk reduction in the ACCOAST trial.[17] It is therefore tempting to speculate that a peri-procedural therapeutic window of platelet inhibition in NST-ACS patients exists as previously suggested in a recent consensus statement.[18]

The present findings suggest that early prasugrel reloading in clopidogrel pre-treated ACS patients who exhibit high platelet reactivity at the time of PCI may provide a more rapid and complete maximum platelet inhibition compared to reloading with 600mg clopidogrel followed by a high dose clopidogrel maintenance dose regimen. The study further indicates that about 40% of patients have high platelet reactivity at the time of PCI despite adequate clopidogrel pre-treatment. This observation corresponds well with previous reports of up to 50% of NSTE-ACS5 and 64.5% of STEMI patients [19] expressing high platelet reactivity after a 600mg clopidogrel-loading dose at the time of PCI.

Whereas the benefit of platelet function test guided antiplatelet therapy for the post-ACS long-term phase has been debated [20,21], there are few studies focusing on this concept in the early, critical phase of an ACS. Bonello investigated the impact of the Vasodilator-stimulated phosphoprotein (VASP) assay and showed that a platelet function testing guided multiple loading regimen was superior to standard therapy in the early ACS phase.[22] Few studies to date have addressed the impact of switching thienopyridine therapy in clopidogrel-pre-treated patients. In healthy subjects, switching from maintenance clopidogrel dosing to prasugrel with additional 60mg loading dose led to a more pronounced platelet inhibition.[23] Likewise, in the Switching Anti Platelet (SWAP) study, switching to prasugrel improved platelet inhibition within 2 hours after additional prasugrel loading dose in patients on clopidogrel maintenance therapy in patients with a previous acute coronary event. However, in the latter study the mean time interval between event and study entry ranged from 77.4 to 102.2 days depending on the treatment arm.[24]

To the best of our knowledge, there are no data from randomised clinical trials investigating the role of platelet function testing using multiple impedance aggregometry in the very early procedural time window in ACS patients. The present results suggest that preselecting patients might help to improve platelet inhibition in this critical phase of an acute coronary event. In the present study, repeated clopidogrel loading dose regimen was not sufficient to overcome high platelet reactivity in ACS patients. This is in line with previous results from pharmacodynamic studies in stable CAD patients. A high dose clopidogrel regimen was only able to reduce high platelet reactivity in non-carriers of the loss-of-function CYP2C19*2 genotype in contrast to prasugrel.[25,26]

## Limitations

This trial has some limitations. First of all it was prematurely terminated due to changing guideline adherence and before the calculated sample size for the primary endpoint was reached. The active comparator of high dose clopidogrel is not favoured by current guidelines. However, due to current treatment patterns and lack of evidence and safety concerns of novel P2Y_12_ receptor antagonist in the upstream period of NSTE-ACS, clopidogrel is still widely used in the ambulance setting and the majority of NSTE-ACS patients in Europe and North America are still pre-treated with clopidogrel. This will likely persist due to higher costs for newer antiplatelet agents. We did not evaluate the impact of the loss- or gain of function genotypes that might have influenced metabolism of the P2Y_12_ receptor antagonists in particular clopidogrel in the acute setting.[27] The concept of genotype-guided antiplatelet therapy is currently debated and not recommended on a routine level.[28,29] The study was not powered for assessment of clinical events and the safety of a platelet function guided approach to reload with prasugrel remains to be investigated in trials that are sufficiently powered to assess for bleeding events. Nevertheless, recent data from a non-randomised, retrospective study supports the feasibility and safety of a prasugrel reloading regimen in ACS patients pre-treated with clopidogrel.[30]

## Conclusions

In conclusion our data provides evidence that residual high platelet reactivity can be corrected by prasugrel in patients with ACS undergoing PCI who have been treated with clopidogrel. The role of platelet function testing is evolving and is not currently recommended as a routine test in guidelines [8,9] while it remains a relevant feature in studies specifically testing antiplatelet response to therapy (e.g. clinicaltrials.gov NCT01959451). Given that clopidogrel continues to be the most commonly used antiplatelet agent in Europe, our data provides further support for clinical studies in health systems that routinely use clopidogrel for the management of ACS. A targeted use of more potent and more effective antiplatelet agents guided by platelet function testing may be warranted with the aim of reducing recurrent ischemic events after ACS, however this should be evaluated in larger randomised studies.

## Supporting Information

S1 CONSORT Checklist The CONSORT statement checklist to improve the reporting of the RCT, enabling readers to understand a trial's conduct and to assess the validity of its results. (PDF)

S1 Protocol Trial protocol describing the study design. (PDF)

S1 Table Table listing Reasons for no recruitment after screening for the APACS- trial. (DOCX)
