# Supplementary material for: High Platelet Reactivity in Patients with Acute Coronary Syndromes Undergoing Percutaneous Coronary Intervention: Randomised Controlled Trial Comparing Prasugrel and Clopidogrel
Source: PLoS One. 2015 Aug 28;10(8):e0135037. doi: 10.1371/journal.pone.0135037 (PMC4552627; doi:10.1371/journal.pone.0135037)
Supplement: S1 Protocol — (PDF) [file pone.0135037.s002.pdf]

Evaluating the benefit of Additional Platelet inhibition in Acute Coronary Syndrome patients with high platelet reactivity undergoing PCI

APACS (HPR) trial

Randomised comparison of prasugrel versus clopidogrel re-loading in patients with ACS undergoing urgent PCI with persistent high platelet reactivity

Protocol Version 5
24th February 2011

EudraCT number: 2010-020219-35

Chief Investigator:
Dr Miles Dalby
Royal Brompton & Harefield NHS Foundation Trust

Study Co-ordination:
Clinical Trials and Evaluation Unit
Royal Brompton and Harefield NHS Foundation Trust

Sponsor:
Royal Brompton & Harefield NHS Foundation Trust

Confidentiality statement
The contents of this document are the property of the Royal Brompton and Harefield NHS Foundation Trust London UK and the Eberhard-Karls University of Tübingen, Germany. The contents may not be used or disclosed in any way without the express permission of the applicants.

SIGNATURE PAGE

Chief Investigator:

Name: Miles Dalby

Title: Consultant Cardiologist

Institution: Royal Brompton & Harefield NHS Trust

Signature: **Date:** 24th Feb 2011

Sponsor Representative:

Name: Wendy Butcher

Title: Head of Governance and Performance

Institution: Royal Brompton & Harefield NHS Trust

Signature: **Date:** 24/2/11

Table of Contents

1 Abbreviations ... 5
2 Contact List ... 6
3 Protocol Summary ... 7
4 Trial Flow Diagram ... 8
5 Trial Flow Chart of Evaluations, Endpoints, Safety ... 9
6 Background ... 10
6.1 Platelet inhibition in acute coronary syndromes ... 10
6.2 Safety of Thienopyridine Re-loading ... 11
6.3 Efficacy and safety of thienopyridine adjustment based on platelet function testing .. 11
6.4 Newer Antiplatelet Agents ... 12
6.5 Role of Platelet Function Guided Therapeutic Approach in Cardiovascular Disease. 13
6.6 Measurement of Platelet Function by Multiple Electrode Aggregometry (“Multiplate”) 14
7 Aims ... 15
8 Study Description ... 15
8.1 Study Design ... 15
8.2 Patient Eligibility Criteria Registry ... 15
8.3 Patient Eligibility Criteria RCT ... 15
8.3.1 Inclusion Criteria ... 15
8.3.2 Exclusion Criteria ... 15
8.4 Screening of Patients ... 16
8.5 Recruitment Rate ... 16
8.6 Informed Consent Procedure ... 16
8.7 Randomisation ... 17
8.8 Randomised Treatment ... 17
8.9 Follow Up ... 17
8.10 Substudies ... 18
9 Study Treatments ... 18
9.1 IMP Regimens ... 18
9.2 IMP Supply and Storage ... 18
9.3 IMP administration ... 18
9.3.1 Day 1 - Randomisation ... 18
9.3.2 Day 2 to day 7 ... 18
9.3.3 Day 8 to day 30 ... 18
9.3.4 Management of High Platelet Reactivity Results ... 19
9.4 IMP Responsibilities and Accountability ... 19
9.5 Post Study Treatment ... 19
9.6 Permitted Concomitant Therapy ... 19
9.7 Contraindicated Concomitant Medical Therapy After Randomisation ... 19
10 Patient Withdrawal ... 19
10.1 Temporary Discontinuation of IMP ... 19
10.2 Permanent discontinuation of IMP ... 19
10.3 Withdrawal from Trial Procedures and Incomplete Follow-up ... 20
11 Trial Observations, Follow Up, Investigations and Tests ... 20
11.1 Case Record Forms (CRF) ... 20
11.2 Time Windows for Follow-up Visits ... 20
11.3 Data to be Collected ... 20
11.4 Adverse/Clinical Endpoints ... 20
11.5 Routine Laboratory Tests ... 20
11.6 Measurement of Platelet Activity ... 21
11.7 Description of Platelet Function Tests and Analysis ... 21
11.8 Blood Samples for Biomarker Substudy ... 21
11.9 Blood Samples for Genetic Substudy ... 21

12 Primary and Secondary Endpoints ... 21
12.1 Primary Endpoint ... 21
12.2 Secondary Endpoints ... 22
12.3 Exploratory Outcomes ... 22
13 Statistical Issues ... 22
13.1 Sample Size ... 22
13.2 Analysis Populations ... 22
14 Regulatory, Ethcal and Legal Issues ... 22
14.1 Regulatory Compliance ... 22
14.2 Ethical Approval ... 23
14.3 Informed Consent ... 23
14.4 Data Protection ... 23
14.5 Source Data ... 23
14.6 Study Records and Archiving ... 23
14.7 Quality Control and Assurance ... 23
14.8 Audits and Inspections ... 24
14.9 Monitoring ... 24
14.9.1 Initiation Visit ... 24
14.9.2 Interim monitoring visits ... 24
14.9.3 Closeout Visit ... 24
15 Pharmacovigilance ... 24
15.1 Safety Reporting ... 24
15.2 Definitions of AEs/ARs/SAEs/SUSARS ... 25
15.2.1 Definition Adverse Event ... 25
15.2.2 Definition Adverse Reactions (AR) ... 25
15.2.3 Definition Serious Adverse Events/Reactions ... 25
15.3 Expectedness of AEs ... 26
15.4 Suspected Unexpected Serious Adverse Reactions (SUSARS) ... 26
15.5 Expected Serious Adverse Events (as a result of the underlying disease or study treatments): ... 26
15.6 Reporting of Serious Adverse Events/Reactions ... 27
15.7 Expedited Reporting of SUSARs ... 27
15.8 Annual Reporting ... 27
16 Trial Organisation and Committees ... 27
16.1 Study Management ... 27
16.2 Trial Steering Committee (TSC) ... 27
16.3 Data Monitoring Committee (DMC) ... 28
16.4 Study Co-ordination ... 28
16.5 Trial Sites ... 28
16.6 Investigators' Responsibilities ... 28
17 End of trial ... 28
17.1 Planned Termination ... 28
17.2 Premature Termination by Sponsor ... 29
17.3 Premature Termination by the Investigator ... 29
18 Publication Policy and Dissemination of Results ... 29
Appendix A: Description of platelet function analyisis (Multi-Plate®) ... 32

1 ABBREVIATIONS

ACS	Acute Coronary Syndrome
AE	Adverse Event
AR	Adverse Reaction
CTEU	Clinical Trials and Evaluation Unit
DMC	Data Monitoring Committee
CYP	Cytochrome P450
ECG	Electrocardiogram
CRF	Case Record Form
IMP	Investigational Medicinal Product
LFT	Liver Function Tests
LD	Loading Dose
MD	Maintenance Dose
o.d.	Once daily
PCI	Percutaneous Coronary Intervention
QP	Qualified Person
RCT	Randomised Controlled Trial
REC	Research Ethics Committee
SAE	Serious Adverse Event
SAR	Serious Adverse Reaction
SD	Standard Deviation
SUSAR	Suspected Unexpected Serious Adverse Reaction
TSC	Trial Steering Committee
VASP	Vasodilator-stimulated phosphoprotein

2 CONTACT LIST

Dr. Miles Dalby<br>Harefield Hospital<br>Hill End Road, Harefield<br>Middlesex UB9 6JH<br>United Kingdom	Tel: +44 1895 828990<br>Fax: +44 1895 828962<br>m.dalby@rbht.nhs.uk	Chief Investigator
Dr. Tobias Geisler<br>Medizinische Klinik III, Department of Cardiology and Cardiovascular disease,<br>Universitätsklinikum Tübingen<br>Otfried Müller Strasse 10<br>72076 Tüebingen<br>Germany	Tel.: +49 7071 2983688<br>Fax: +49 7071 295749<br>tobias.geisler@med.uni-tuebingen.de	Principal Investigator
Dr Azfar Zaman<br>Freeman Hospital and University of Newcastle,<br>Newcastle-upon-Tyne NE7 7DN<br>United Kingdom	Tel: +44 191 213 7277<br>Fax: +44 191 223 1417<br>azfar.zaman@nuth.nhs.uk	Principal Investigator
Wendy Butcher<br>Research Office<br>Royal Brompton and Harefield NHS Foundation Trust,<br>Sydney Street,<br>London SW3 6NP<br>United Kingdom	Tel: +44 0 207 351 8109<br>Fax: +44 207 351 8578<br>w.butcher@rbht.nhs.uk	Sponsor
Marcus Flather<br><br>Jean Booth<br><br>Clinical Trials and Evaluation Unit<br>Royal Brompton and Harefield NHS Foundation Trust<br>Sydney Street<br>London SW3 6NP<br>United Kingdom	Tel: +44 207 351 8827<br>m.flather@rbht.nhs.uk<br><br>Tel: +44 207 351 8864<br>j.booth@rbht.nhs.uk<br><br>Fax +44 207 351 8829	Clinical Trials & Evaluation Unit

3 PROTOCOL SUMMARY

**Title**	Evaluating the benefit of additional platelet inhibition in acute coronary syndrome patients with high platelet reactivity undergoing PCI
**Acronym**	APACS-HPR Trial
**Study Aim**	Does reloading with prasugrel in acute coronary syndrome (ACS) patients undergoing urgent PCI who respond poorly to clopidogrel lead to improved periprocedural platelet inhibition compared to clopidogrel reloading
**Study Design**	1. Screening registry of potentially eligible patients<br>2. Multi-centre, randomised open-label clinical trial
**Eligibility Criteria**	Inclusion criteria:<br>• Acute coronary syndromes UA/(NSTEMI/STEMI) with intention to perform , PCI <72 hours from onset of symptoms that led to hospital admission,<br>• Platelet reactivity >400Au/min as measured by multiplate testing device ≥ 2 hrs from first (non-study) loading dose clopidogrel<br>Main Exclusion criteria:<br>• Increased risk of major bleeding<br>• Prior or planned GPIIb-IIIa inhibitor treatment<br>• Known contraindications to thienopyridines
**Treatments**	Group 1: clopidogrel repeat loading dose (600mg) followed by 150mg clopidogrel once daily for up to 7days, followed by 75mg clopidogrel once daily<br>Group 2: prasugrel loading 60mg followed by 10mg once daily. for 30 days;
**Sample size**	N=70 per arm (total 140)
**Randomisation**	1:1 randomisation
**Primary and secondary endpoints**	**Primary**:<br>Proportion of patients with improved platelet response (i.e. decreased platelet reactivity under the cut-off value of 400 Au.min) in the prasugrel re-loading arm compared to the clopidogrel re-loading arm at 4 hours after randomization in patients with initial high platelet reactivity<br>**Secondary**:<br>• Area under the curve for CK, troponin<br>• Clinical events (MACE) and bleeding
**Recruitment Period**	18 months
**Follow Up Period**	30 days
**Sponsor**	Royal Brompton and Harefield NHS Foundation Trust

4 TRIAL FLOW DIAGRAM

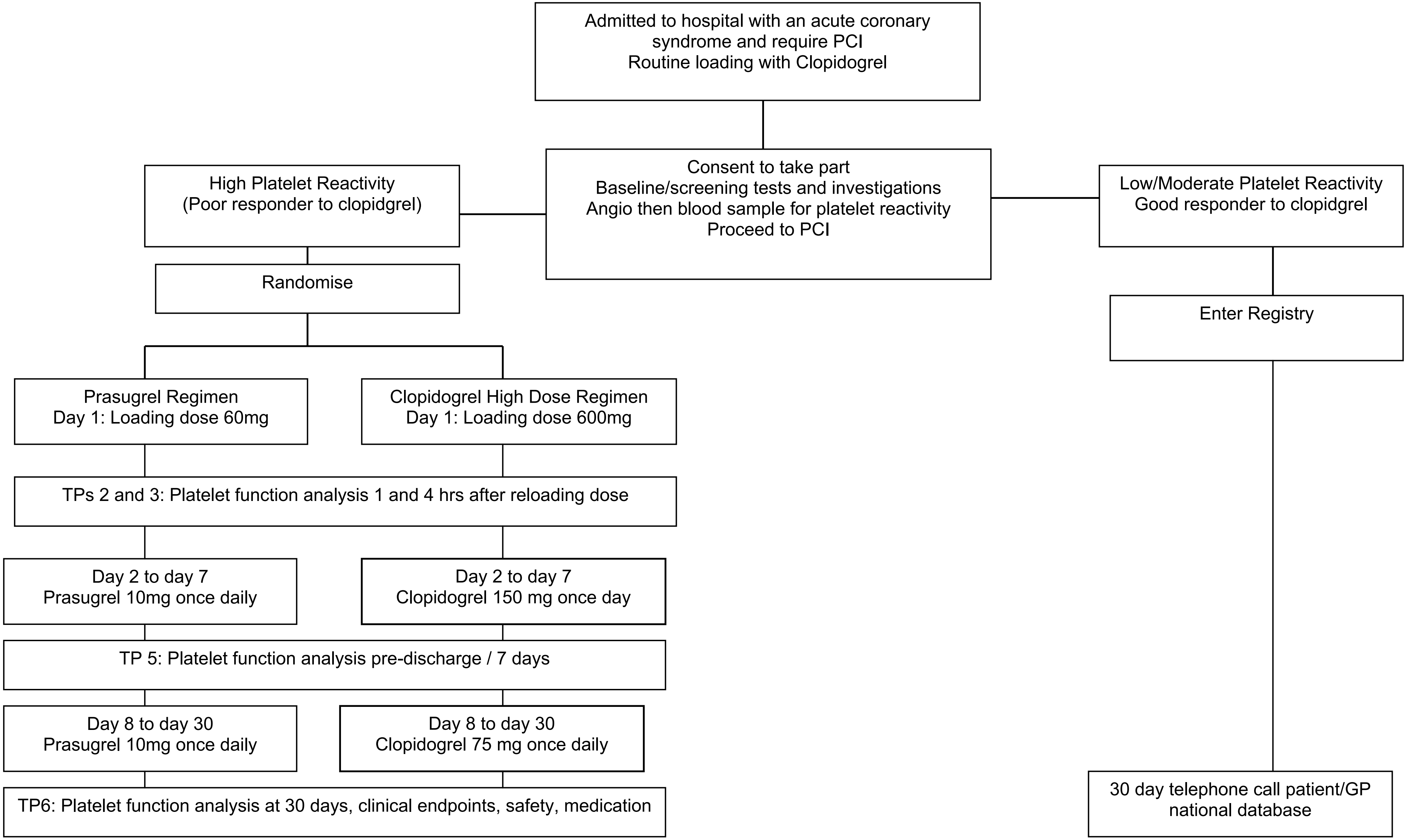

5 TRIAL FLOW CHART OF EVALUATIONS, ENDPOINTS, SAFETY

Investigation	TP0 Baseline	TP 1 Angio/PCI	TP 2 (1 hr > reloading)	TP 3 (4hr > reloading)	TP 4 (24hr > reloading)	TP 5 (pre discharge)	TP 6 (day 30)
Informed consent	X Δ						
Eligibility criteria	X Δ						
Demographics	X Δ						
Medical history	X Δ						
Physical exam	X Δ						
Routine haematology	X Δ						X
Routine clinical chemistry	X Δ						X
CK, troponin I	X Δ		X	X	X		
Pregnancy test*	X Δ						
Endpoint							
Platelet activity		X Δ	X	X		X	X
Clinical endpoints						X	X Δ
Substudies							
Biomarker blood sample	X Δ				X	X	
Genetic blood sample	X Δ						
Treatment							
Concomitant medication						X	X
Compliance						X	X
Safety							
AEs			X	X	X	X	X

TP = time point
* = pregnancy test if appropriate
X= RCT patients
Δ = Registry patients
TP1 = This blood sample must also be at least 2 hours after loading with clopidogrel.

6 BACKGROUND

6.1 Platelet inhibition in acute coronary syndromes

Acute coronary syndromes (ACS) are associated with a high morbidity and mortality. Several treatments including dual antiplatelet therapy (DAPT) consisting of aspirin and an Adenosin-Diphosphate (ADP)-Receptor blocker reduce the risk of subsequent vascular events including myocardial infarction and stent thrombosis. Some of the benefits of DAPT for ACS patients undergoing PCI appear to be related to pre-treatment with a loading dose of the ADP-receptor blocker clopidogrel.[1] However, in a substantial proportion of patients who undergo urgent PCI an optimal pre-loading with clopidogrel cannot be guaranteed in real world clinical practice and even if optimal pre-treatment is provided, other mechanisms can lead to decreased antiplatelet efficacy like clinical risk factors and genetic polymorphisms.

We recently found a number of distinct clinical variables to be associated with reduced and attenuated response to antiplatelet therapy with clopidogrel [2,3] Genetic "loss-of-function" variants (polymorphisms) especially of the 2C19 isoenzyme of cytochrome P450 have been proposed to cause decreased responsiveness to clopidogrel.[4,5] Newer antiplatelet substances targeting the ADP-receptor (prasugrel, ticagrelor) on platelets are less susceptible to this loss of function genotype due to different metabolization pathways.[6,7] The degree of peri-interventional residual platelet aggregation correlates with the occurrence of short-term cardiovascular events.[8,9,24] There is a direct relationship between the degree of peri-interventional platelet aggregation and the risk of stent thrombosis, a serious cardiac event which is associated with up to 50% mortality. Therefore, we could observe that a high residual platelet aggregation is associated with up to 100-fold relative change in baseline hazard for risk of stent thrombosis (figure 1).

Recent data indicate that additional platelet inhibition (i.e. by higher loading and maintenance dosing of clopidogrel) leads to improved outcome in ACS patients treated by PCI.[10] Additionally, thienopyridine loading beyond 600-mg clopidogrel provides higher platelet inhibition with a tolerable safety profile[11,12,13.] The results of the recently published ARMYDA-4 RELOAD trial showed that re-loading with clopidogrel before PCI in patients already on thienopyridine treatment did not show effects on the primary composite endpoint of 30-day death, myocardial infarction, and target vessel revascularization in the overall study population, however revealed a significant favourable effect in the subgroup of ACS patients.[14] In the light of these previous hypothesis-generating observations, achievement of optimal and timely platelet inhibition during coronary intervention in ACS-patients is essential to reduce atherothrombotic risk and the effects of additional P2Y12 receptor blockade on behalf a platelet function analysis should be further evaluated.

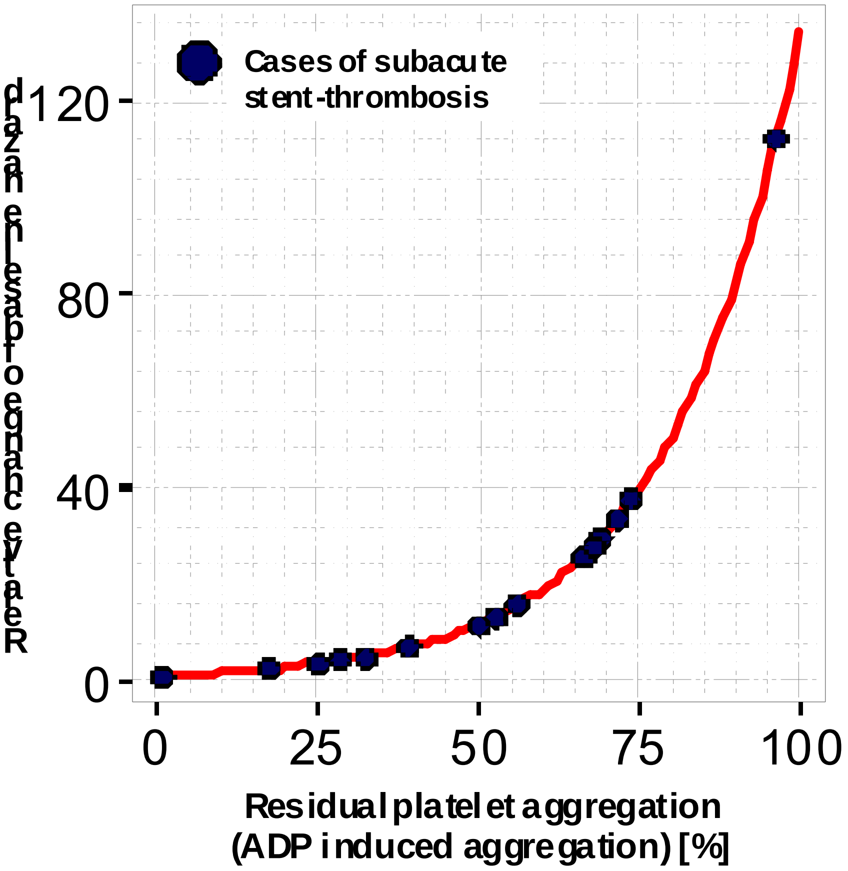

Figure 1: Relationship of the risk (Hazard) for the occurrence of subacute stent-thrombosis depending on peri-interventional ADP-induced aggregation, analysis of 1019 patients (according to Geisler T, et al, Eur Heart 2010)

6.2 Safety of Thienopyridine Re-loading

Additional loading beyond 600mg clopidogrel leads to a higher degree and faster onset of platelet inhibition with a tolerable safety profile.[11,12,13] In the RELOAD study, a repeated loading with 900mg clopidogrel in patients already pre-treated with clopidogrel improved platelet inhibition without causing an excess in TIMI major bleedings.[15]

6.3 Efficacy and safety of thienopyridine adjustment based on platelet function testing

Recently, there have been approaches to adjust thienopyridine loading on behalf the results of platelet function testing. In two studies, Vasodilator-stimulated phosphoprotein (VASP) assay was applied to tailor clopidogrel loading in ACS patients undergoing PCI. Patients with a VASP index > 50% were re-loaded up to a total dose of 2,400mg. This regimen was able to reduce short-term major cardiovascular events including stent thrombosis compared to patients without VASP guided treatment and did not cause a relevant increase in bleeding risk.[13,16] However, platelet reactivity could not be converted to a degree of adequate responsiveness in up to 14% of the patients, suggesting that these patients do not respond to clopidogrel and may benefit from alternative P2Y12 receptor inhibition. The VASP assay cannot easily be transferred into clinical routine due to the

requirement of a quality assured platelet function lab, well trained laboratory assistants and is unsuitable for point-of-care decision making due to time consuming laboratory procedure and lack of standardisation of the method. Furthermore, in the acute setting of ACS it is not useful as it cannot provide immediately available results. Therefore, the effects of instantaneous therapy adjustment in ACS patients undergoing primary PCI have to be further evaluated by rapid platelet function assays.

6.4 Newer Antiplatelet Agents

Prasugrel is a 3rd generation thienopyridine that, like clopidogrel, needs biotransformation into its active compound. However, the differences in metabolism allow for greater and faster bioavailability of prasugrel's active metabolite compared to clopidogrel's. A major part of clopidogrel is inactivated by esterases and only a smaller part is further metabolized by two cytochrome P450 (CYP) dependent oxidization steps to its active metabolite.[17] Conversely, prasugrel is partly activated by esterases and only a single CYP metabolization step is necessary for conversion into the active form.[18] Therefore prasugrel achieves greater levels of active metabolite formation and a faster onset of platelet inhibition[19] (figure 2) which can be advantageous when rapid platelet inhibition is demanded in acute coronary events.

In the Trial to Assess Improvement in Therapeutic Outcomes by Optimizing Platelet Inhibition with Prasugrel - Thrombolysis in Myocardial Infarction (TRITON-TIMI 38) the effectiveness of prasugrel was proven for the treatment of patients with ACS undergoing PCI. Thirteen thousand six hundred and eight patients with either STEMI (26%) or unstable angina and non-STEMI (74%) were randomised to prasugrel (60 mg LD, 10mg daily MD) or clopidogrel (300 mg LD, 75 mg daily MD) for a median duration of 14.5 months. There was a significant reduction of the primary endpoint (CV death, non-fatal myocardial infarction and stroke) in the prasugrel treated arm (9.9% versus 12.1% in the clopidogrel arm, P<0.001; number needed to treat 46). Additionally, stent thrombosis was decreased by 50% in prasugrel treated patients which was mainly due to a reduction of early stent thromboses. On the other side, there was a significantly higher incidence of TIMI major, non-CABG associated bleedings (2.4 versus 1.8%, p=0.03) and life-threatening bleeds with fatal outcome (0.4 versus 0.1%; p=0.002), that was most marked in subgroups of patients with a previous cerebrovascular event, older patients (≥75 years) and patients with a body weight of less than 60kg. The clinical net benefit (TIMI major bleeding plus CV death, non-fatal myocardial infarction and stroke) remained in favour of prasugrel treatment, except in those subgroups.[20] According to recent studies, the metabolism and action of prasugrel seems not to be affected by CYP polymorphisms. A retrospective analysis of the TRITON-TIMI 38 cohort revealed no effects of SNPs in the genes for CYP2C19, CYP2C9, CYP2B6, CYP3A5 or CYP1A2 in the prasugrel treated arm, however, efficacy of clopidogrel was reduced in carriers of polymorphic CYP2C19 loss-of-function variants.

The US Food and Drug Administration (FDA) approved prasugrel for clinical use based on the TRITON data, stating that the efficacy of prasugrel outweighs the bleeding risk due to the low frequency of fatal bleedings. At the same time, they recommended that prasugrel should be considered after careful assessment of the individual bleeding risk.[21] In the UK the National Institute of Health and Clinical Excellence (NICE) has supported the use of prasugrel for STEMI-patients, diabetics with ACS undergoing PCI and those with stent thrombosis (National Institute of

Health and Clinical Excellence.[22] In comparison, the non-thienopyridine ADP-receptor ticagrelor antagonist has been documented superior platelet inhibition compared to clopidogrel and was associated with a significant reduction of the primary endpoint (CV death, MI and stroke) and a tolerable safety profile in ACS patients compared to standard clopidogrel treatment in the PLATO trial.[23] Although, ticagrelor has been assigned a similar recommendation level for use in ACS patients undergoing percutaneous coronary intervention (see ESC guidelines for myocardial revacularization 2010), there are to date less established practical guidelines how to include this promising substance in the treatment algorithm for ACS. Additionally, compliance issues caused by necessary two time daily dose have not been sufficiently addressed and investigated in real world clinical practise. Therefore, the current protocol focuses to investigate only the effects of additional prasugrel adjustment guided by platelet function testing approach in ACS patients.

6.5 Role of Platelet Function Guided Therapeutic Approach in Cardiovascular Disease

In the Gauging Responsiveness With A VerifyNow Assay-Impact On Thrombosis And Safety GRAVITAS trial, 5429 patients on the regular clopidogrel dose underwent platelet-function tests with the VerifyNow assay (Accumetrics, San Diego, CA) 12 to 24 hours after PCI. Of these, 2214 (41%) had high residual platelet reactivity (platelet reactivity units [PRU] >230) and were randomised to continue on the 75-mg regular clopidogrel dose or to receive another 600-mg loading dose and a higher maintenance dose of 150 mg daily. There was no difference in the primary endpoint in between patients with high PRU who received either intensified dosing or standard therapy. At six months of follow-up, the composite end point of cardiovascular death/MI/stent thrombosis was identical at 2.3%.The absolute platelet reactivity was only modestly reduced from an average of about 280 PRU at baseline to 200 PRU in the high-dose clopidogrel group vs 240 PRU in the standard-dose group (Price MJ, presented at the congress of the American Heart Association 2010). The key issues of the trial can be summarized as followed: in stable patients there is no clinical benefit of increasing clopidogrel dosing in patients with high platelet function measured by the VerifyNow assay. Additionally, there is no relevant pharmacodynamic effect of high clopidogrel dosing measured by platelet function analysis in the stable setting. From the background of this trial, there is further need to evaluate the role of a platelet function guided approach in the ACS setting which is associated with higher and more variable pretreatment platelet aggregation. Additionally, application of more potent platelet inhibitors like prasugrel may contribute to a larger effect size leading to more pronounced pharmacodynamic and clinical effects.

The “Testing Platelet Reactivity In Patients Undergoing Elective Stent Placement on Clopidogrel to Guide Alternative Therapy With Prasugrel” (TRIGGER)-PCI Trial is recruiting patients to evaluate the effects on clinical outcome after randomization to either prasugrel or clopidogrel in stable DES treated patients having a high platelet reactivity as measured with the VerifyNow Assay (clinicaltrials.gov NCT00910299). However, this point-of-care assay is not affordable to every interventional centre in a routine fashion due to high consumable costs. Thus other near patient testing methods in acute coronary syndromes should be evaluated for their impact on therapeutic adjustment with alternative P2Y12 inhibitor treatment.

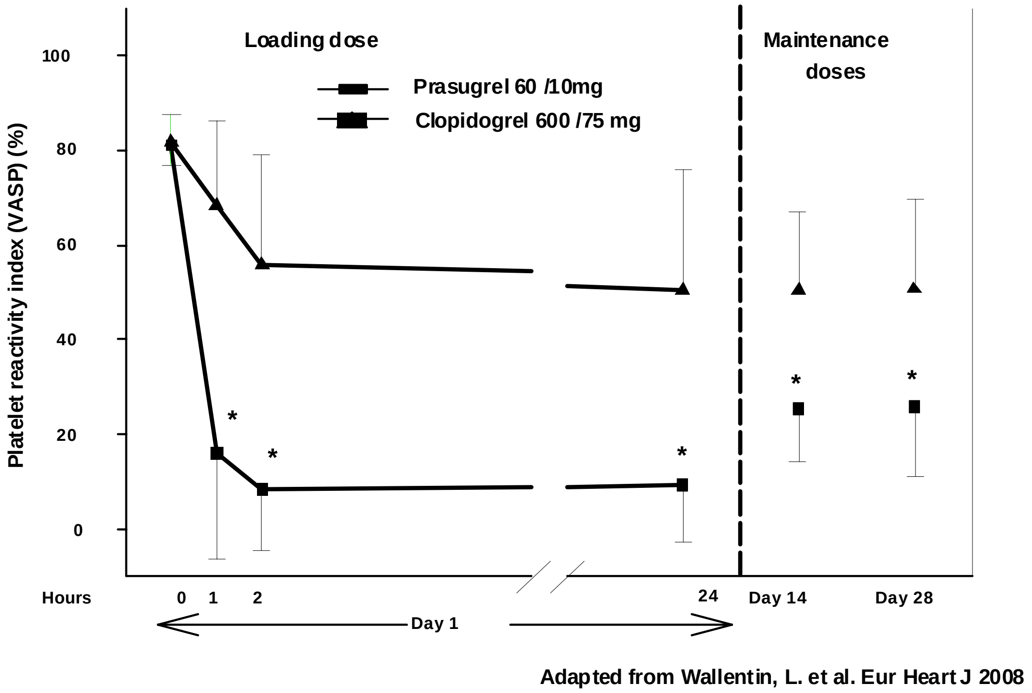

Figure 2: Higher degree and more rapid onset of platelet inhibition by prasugrel compared to clopidogrel

6.6 Measurement of Platelet Function by Multiple Electrode Aggregometry ("Multiplate")

In recent years there has been cumulating data provided by several groups indicating that this rapid platelet function assay is able to detect effects of thienopyridine treatment and to correlate laboratory results to clinical prognosis in patients undergoing PCI.[24,25,26] Due to short sample preparation, easy installation in the cath lab and simple and standardized, semi-automated application, this assay is especially suitable for the setting of the planned study involving acute coronary syndrome patients undergoing primary PCI.

We are proposing to identify the proportion of patients with high platelet reactivity who have are poor responders to a standard loading dose of Clopidogrel in ACS patients who require urgent PCI. We will evaluate a prasugrel re-loading regimen versus a clopidogrel reloading high dose regimen in a randomised clinical trial. Screened patients identified with low platelet reactivity who are good responders to clopidogrel will be entered into a Registry.

7 AIMS

1. Perform a screening registry in consecutive ACS patients undergoing urgent PCI to determine the proportion that are poor responders to clopidogrel defined as high platelet reactivity
2. Investigate efficacy and feasibility of a platelet function guided approach for antiplatelet drug adjustment in ACS patients undergoing PCI
3. Investigate additional antiplatelet effects of prasugrel reloading compared to clopidogrel reloading in ACS-patients with a high ADP-induced platelet aggregation ("poor responders")
4. Document and compare proportion of patients who persist with high RPR before hospital discharge
5. Evaluate the safety of early reloading with antiplatelet therapy in patients with high RPR (poor responders)

8 STUDY DESCRIPTION

8.1 Study Design

This is a multinational, randomised open label study comparing prasugrel versus clopidogrel in ACS patients who have high platelet reactivity managed with an early invasive strategy (i.e. intention to perform PCI as early as possible and no later than 72 hours from admission).

Screened patients identified with low platelet reactivity indicating a good response to clopidogrel will be entered into a Registry.

8.2 Patient Eligibility Criteria Registry

a) ACS patients with intent for PCI <72 hours from admission.
b) Prior clopidogrel loading within 24h before planned PCI or chronic (>24 hours) treatment with clopidogrel
c) Low platelet reactivity (HPR) PA < 400 AU min by multiplate analyser ("good responders"). Blood sample to be taken at least 2 hrs after prior clopidogrel loading.
d) Consent.

8.3 Patient Eligibility Criteria RCT

8.3.1 Inclusion Criteria

a) ACS patients with intent for PCI <72 hours from admission.
b) Prior clopidogrel loading within 24h before planned PCI or chronic (>24 hours) treatment with clopidogrel
c) High platelet reactivity (HPR) PA ≥ 400 AU min by multiplate analyser ("poor responders").
d) Initial platelet function sample at least 2 hours after pre PCI loading dose
e) Consent.

8.3.2 Exclusion Criteria

a) Patients <18 years and >75 years
b) Body weight <60kg

c) Pretreatment with prasugrel within 7 days of randomisation
d) History of stroke or transient ischaemic attack
e) Patients with increased bleeding risk e.g.
    - recent major trauma or surgery
    - gastrointestinal bleeding or active peptic ulceration
    - Platelet count <100,000 / mm^3 at the time of screening
    - Internationally Normalized Ratio (INR)> 1.5 at the time of screening
f) Hb<10g/dL
g) Intracranial neoplasm, arteriovenous malformation or aneurysm.
h) Severe hepatic impairment (Child Pugh class C)
i) Intention to use the following medications
    - oral anticoagulation
    - other antiplatelet therapy (including GPIIb/IIIa inhibitors) besides aspirin
    - nonsteroidal anti-inflammatory drugs (NSAIDs) or cyclooxygenase-2 (COX-2) inhibitors
j) Female patients who are pregnant, planning pregnancy, not using reliable contraception or who are breastfeeding
k) Known allergy, hypersensitivity or other contraindications to prasugrel or clopidgrel

8.4 Screening of Patients

All patients admitted to the hospital with suspected ACS (unstable angina, NSTEMI or STEMI) requiring urgent PCI will be screened for entry into the trial by the investigator or his/her designee. Patients will be assigned a screening number and the investigator or his/her designee will explain the study requirements and procedures and obtain consent before any study procedures are performed.

A study blood sample will be required at the end of the angiogram before proceeding to PCI (±30 mins from start of the procedure) to evaluate platelet activity. This blood sample must also be taken at least 2 hours after prior loading with clopidogrel. If this is not achievable for clinical reasons then this patient would not be eligible for the study. Blood analysis for platelet activity will take about 20 minutes. Those patients who are poor responders to non-study loading dose of Clopidogrel defined as high platelet activity PA $\geq$ 400 AU min will be eligible for randomisation. The good responders to Clopidogrel (PA< 400AU min) will be entered into a registry and baseline data, platelet activity results recorded onto the registry CRF.

8.5 Recruitment Rate

We estimate approximately 500 patients will be screened. From these a total of 140 patients will be randomised, the remaining screened patients will be entered into the registry. There are 3 sites participating, recruitment will be competitive recruitment and each site is expected to randomise about 40 to 50 patients each. We estimate 7 to 8 patients randomised per month over a period of 18 months.

8.6 Informed Consent Procedure

Patients who are admitted and require an urgent PCI, such as STEMI or high risk unstable NSTEMI, will be requested to provide verbal assent to enter the Registry and if eligible, the randomised controlled trial (RCT). The assent process involves reading an ethically approved short narrative of the study to the patient and if the patient

provides verbal agreement to enter the study (assent), this will be documented in the hospital record. Within 24 hours of the PPCI procedure, assuming the patient's clinical condition allows, full written informed consent will be obtained for the registry and/or RCT as appropriate. The assent process has been approved on ethical grounds for patients with critical conditions who are participating in clinical trials. Thus patients will be required to provide verbal assent and written informed consent in order to participate fully in the study.

Patients who are stable and able to have written informed consent prior to screening procedures will do so. These patients will be required to provide written informed consent to enter the Registry and if eligible the RCT.

The process of consent requires individual discussion with the patient. Information should be provided in a language and at a level of complexity understandable to the subject in both oral and written form. Patients should not be coerced, persuaded, or unduly influenced to participate or remain in the trial. Patients should understand that they are free to withdraw from the trial at any point and that this decision will not affect the level of care they will receive. Before any trial-related procedures may be performed, assent or written informed consent must be obtained from the patient by the investigator or hi/her designee. For patients who initially give assent written informed consent must be obtained for the patient to be able to continue in the study (either registry or RCT). If patients provide verbal assent but not subsequent written consent, patients are not able to proceed in the study. Data already gathered may only be used if patients provide consent for this purpose, even if they do not consent to continuing in the study.

8.7 Randomisation

Randomisation blocks will be generated by a statistician. The investigator or his/her designee will access either a web based randomisation service or a telephone Interactive randomisation service. Randomisation will occur at the end of the PCI procedure as soon as the platelet activity results are known. The investigator or his/her designee will access the randomisation service most likely from the catheter laboratory or recovery/ward area. Confirmation of eligibility and written consent will be required from the investigator or his/her designee. A randomisation allocation either clopiogrel regimen or prasugrel regimen will be released. Each randomised patient will be assigned a unique identifying number. Confirmation of randomisation will be sent to the co-ordinating centre.

8.8 Randomised Treatment

Randomised patients will be allocated to receive either open label Clopidogrel (Plavix) or Prasugrel.

8.9 Follow Up

Patients in the RCT will be required to attend the hospital for a follow up visit at 30±3 days from date of randomisation. Patients will be assessed by the investigator or his/her designee for vital status, medication tolerance and compliance, occurrence of adverse events and clinical endpoints.

Patients in the Registry will be have 30 day follow up for vital status either by telephone call to the patient, their general practitioner or by checking with national databases.

8.10 Substudies

Sub-studies of high scientific merit will be conducted based on the recommendation of the Trial Steering Committee and will have received ethical approval.

9 STUDY TREATMENTS

9.1 IMP Regimens

Randomised patients will be allocated to receive either open label clopidogrel (Plavix) or prasugrel.
Group 1: Clopidogrel (Plavix)

- Day 1 Loading 600mg
- Day 2 to 7 day: 150mg o.d.
- Day 8 to 30 days: 75mg o.d.

Group 2: Prasugrel

- Day 1 loading 60mg
- Day 2 to 7 10mg o.d.
- Day 8 to 30 days 10mg od

9.2 IMP Supply and Storage

Prasugrel, a commercially available product, will be supplied by Eli Lilly which holds the manufacturing license to produce the IMP. Clopidogrel (Plavix) will be purchased by the hospital Pharmacy through normal purchasing arrangements. All study drugs should be stored in an appropriate locked room, under the control of the Hospital Pharmacist or the Investigator, in the conditions described in the package insert.

9.3 IMP administration

9.3.1 Day 1 - Randomisation

Immediately after randomization (Day 1) the loading dose of the study drug must be given as early as possible. Patients in the Clopidogrel arm will be required to take 8 x75mg tables = 600mg and patients in the prasugrel arm will be required to take 6 x 10mg = 60mg. Once the loading dose is administered, the person who administered the drug must sign the drug chart confirming the patient successfully swallowed all administered tablets. Any deviations or problems must be recorded.

9.3.2 Day 2 to day 7

- Patients in the clopidogrel group will receive 150mg (2x75mg tablets) of open label clopidogrel study drug.
- Patients in the prasugrel group will receive 10mg (1 tablet) of open label prasugrel.

9.3.3 Day 8 to day 30

- Patients in the clopidogrel group will receive 75mg (1 tablet) of open label Clopidogrel study drug.

- Patients in the prasugrel group will receive 10mg (1 tablet) of open label prasugrel.

9.3.4 Management of High Platelet Reactivity Results

The investigators will be aware of the platelet activity results. Patients who continue to have high platelet reactivity results will be reviewed by the investigator. The decision on subsequent antiplatelet therapy will be at the discretion of the investigator. Details of any cross over will be captured in the case report form and reviewed.

9.4 IMP Responsibilities and Accountability

The Investigator, the Hospital Pharmacist, or other personnel allowed to store and dispense IMP will be responsible for ensuring that the IMP used in the clinical trial is securely maintained in accordance with the applicable regulatory requirements. All IMPs shall be dispensed in accordance with the Investigator's prescription, and it is the Investigator's responsibility to ensure that an accurate record of IMP issued and returned is maintained. A treatment log of the returned IMP will be established with the Investigator or the Hospital pharmacist. Accurate records will be kept of destroyed product. It is recommended that, unless clear contra-indications arise, study drug be continued or only briefly interrupted.

An appropriate record of study drug accountability, as well as a record of all pre-specified concomitant medications must be kept at each scheduled visit.

9.5 Post Study Treatment

Post study treatment will depend on the patient’s physician. Patient may continue on open label clopidogrel or prasugrel as decided by the patient’s physician.

9.6 Permitted Concomitant Therapy

All patients should receive aspirin 75-150mg once daily.

9.7 Contraindicated Concomitant Medical Therapy After Randomisation

Oral anticoagulants, oral dipyridamole, and open-label use of thienopyridines (clopidogrel or ticlopidine) are not allowed concomitantly with study drug administration. If such a treatment becomes necessary, then the study drug should be temporarily interrupted. Investigators should restart study drug as soon as possible after such concomitant treatment is stopped. No other investigational drugs are allowed for the duration of the study.

10 PATIENT WITHDRAWAL

10.1 Temporary Discontinuation of IMP

Temporary discontinuation of drug is discouraged unless for adverse event or side effects. In such cases, the Investigator should down titrate or discontinue the study medication as required. Reintroduction of study medication should be monitored

10.2 Permanent discontinuation of IMP

Permanent IMP discontinuation is only clearly justified for an adverse event /or for bleeding or when the qualifying condition is not present. In other situations,

discontinuations will be discouraged as much as possible. Patients may permanently withdraw from treatment with the IMP if they decide to do so, at any time and for any reason, or this may be the Investigator's decision. If there is considered to be a concern about the batch of IMP, recall procedures will be in place and measures will be taken to ensure the safety of all trial participants.

10.3 Withdrawal from Trial Procedures and Incomplete Follow-up

Patients are free to withdraw consent from trial procedures at any time. Investigators must ascertain the reasons for the withdrawal, including discontinuation of study drug, withdrawal from study investigations and/or follow up, withdrawal due to adverse events, failure to attend, non-compliance, withdrawal of consent or other reasons. CTEU must be notified of withdrawals within 5 working days, unless withdrawal is due to a SAE, in which case the investigator will follow SAE reporting procedures.

Withdraw from trial procedures may result in incomplete patient follow-up and failure to capture outcome data. In these cases as much data as possible will be collected up until the point of withdrawal. Patients may choose to withdraw from trial procedures and request that further data are not collected.

11 TRIAL OBSERVATIONS, FOLLOW UP, INVESTIGATIONS AND TESTS

11.1 Case Record Forms (CRF)

Trial data will be captured on a case record form (CRF). The CRF will be designed in accordance with the requirements of the trial protocol and will comply with regulatory requirements.

11.2 Time Windows for Follow-up Visits

There is one follow up visit at 30 days from the date of randomisation. Every effort must be made to see patient at 30days. An additional 5 days is permitted in exceptional circumstances

11.3 Data to be Collected

A full list of data to be collected will be provided as an appendix. A flow chart with the schedule of evaluations is provided on page 9.

11.4 Adverse/Clinical Endpoints

The checking for the occurrence of adverse events and clinical endpoints will begin from randomisation and will continue for the individual patient until they complete their follow up at 30 days. Details of adverse and clinical events will be captured on specific case report forms.

11.5 Routine Laboratory Tests

Laboratory test results must be obtained by local routine methods. Local normal values will be collected and the investigator must notify CTEU of any subsequent changes in normal values. A copy of the computer print-out with laboratory test results, showing the current local normal ranges may be provided to CTEU. Abnormal results need to be commented upon by the investigator as regards clinical relevance.

11.6 Measurement of Platelet Activity

A 5ml blood sample will be taken for platelet function analysis using the Multiplate©-Impedance aggregometry.

Investigation time point (TP)	Patient population	Timing of test
TP 1	All screened patients	>2 hours of initial (non-study) loading of clopidgrel and ±30min of start of PCI
TP2	Randomised patients	1 hrs post study drug loading
TP3	Randomised patients	4 hrs post study drug loading
TP4	Randomised patients	24 hrs post study drug loading
TP5	Randomised patients	day 7 or hospital discharge whichever occurs earlier
TP6	Randomised patients	30 follow up

11.7 Description of Platelet Function Tests and Analysis

Details for platelet activity analysis are provided in Appendix A.

11.8 Blood Samples for Biomarker Substudy

Approximately 10ml of blood will be taken for the biomarker substudy at baseline (TP0) from all patients (registry and RCT). Additional samples (10ml) will be obtained at TP4 and 5 in randomised patients only. These samples will be stored locally in a freezer and shipped at regular intervals to a core lab based in Germany.

11.9 Blood Samples for Genetic Substudy

Approximately 15ml of blood will be taken for the genetic substudy at baseline from all patients (registry and RCT). This sample will be stored locally in a freezer and shipped at regular intervals to a core lab based in Germany.

Genetic testing is a part of this study. Blood samples for directed genomic testing will be collected at TP0. DNA derived from these samples will be used to determine the influence of genetic variants on treatment response, metabolism, action, or AEs. These evaluations may include genetic analysis of drug transporter, metabolizing enzymes such as CYP450 and esterases, and/or P2Y12 receptor polymorphisms. These samples will retain the subject identifier and, therefore, will not be stored indefinitely, but will be destroyed within 3 years after the last subject visit for the study.

12 PRIMARY AND SECONDARY ENDPOINTS

12.1 Primary Endpoint

The primary endpoint will compare the proportion of patients with improved platelet response (i.e. decreased platelet reactivity under the cut-off value of 400 Au.min) in the prasugrel re-loading arm compared to the clopidogrel re-loading arm at 4 hours after randomization in patients with initial high platelet reactivity

12.2 Secondary Endpoints

- To compare the proportion of patients with improved platelet response between the treatment arms at hospital discharge/7 days and at 30 days.
- To compare the AUC for CK and troponin at 24 hours between the treatment arms
- To compare the rates major adverse events (death, myocardial infarction, stroke, repeated revascularization) at 30 days between the treatment arms
- To compare the rate of major bleedings at 30 days between the treatment arms.

12.3 Exploratory Outcomes

Explorative objectives include the conversion rate to adequate platelet inhibition by switching patients with persistent HPR after clopidogrel reloading to prasugrel maintenance therapy.

13 STATISTICAL ISSUES

13.1 Sample Size

For the pilot study if we expect an absolute difference of 20% in conversion of patients with initial HPR to a platelet reactivity degree under the cut-off value (i.e. reduction of prevalence from 30% to at least 10%) after re-loading with prasugrel compared to clopidogrel the estimated minimum sample size would be 62 patients per arm (80% power at a two-sided alpha value of 5%) to detect this difference.

13.2 Analysis Populations

Intention to Treat
The intention to treat (ITT) population is defined as all patients randomised to treatment regardless of compliance. All patients will be analysed according to the treatment they were allocated to. All analyses will be carried out using the ITT population.

Per Protocol
A per-protocol analysis will be considered if there is considerable number of protocol violators. This decision will be made jointly by the trial statistician in co-operation with members of the Trial Steering Committee on examination of the population. The decision will be made without reference to the endpoint data and all analyses will be summarised in the Statistical Analysis Plan (SAP) prior to data base lock.

14 REGULATORY, ETHCAL AND LEGAL ISSUES

14.1 Regulatory Compliance

This study will comply with the European Clinical Trials Directive and the International Conference on Harmonisation Good Clinical Practice (ICH-GCP) guidelines. Applications will be made for a clinical trials authorisation (CTA) to each applicable regulatory authority prior to starting the study (e.g. MHRA in UK and BfARM in Germany). The study will be registered in the European Community with a EudraCT number. Protocol amendments must be approved by the regulatory authority. SUSAR reports, annual safety updates and notification of the end of the study will be sent to the regulatory authorities.

14.2 Ethical Approval

The trial will comply with the Declaration of Helsinki on research involving human subjects. The study protocol, patient information sheet(s) and informed consent forms (ICF) will be submitted to the relevant Research Ethics Committees in each participating country for approval and institutional approval will be sought where applicable. A signed Clinical Trial Agreement with each of the centres will be required before the study commences. Protocol amendments must be approved by the Research Ethics Committee. Annual reports and notification of the end of the study will be sent to the Research Ethics Committee.

14.3 Informed Consent

All patient information sheets, consent forms will be approved by the regulatory authorities and the research ethics committee and any subsequent amendments. The Investigator (according to applicable regulatory requirements), or a person designated by the Investigator, should fully inform the patient of all aspects of the clinical trial. Prior to a patient's participation in the clinical trial, the Informed Consent Forms should be signed and personally dated by the patient or by the patient's legally acceptable representative, and by the nominated person who conducted the informed consent discussion.

14.4 Data Protection

All personnel involved in the trial will work within the confines of the local data protection regulations.

A unique identification code assigned to each subject will be used in lieu of the subject's name to protect the subject's identity when reporting AEs and/or other study-related data.

14.5 Source Data

For all study procedures, relevant clinical findings, procedures performed and changes in medication must also be documented in the patient's hospital file.

14.6 Study Records and Archiving

The Investigator must maintain confidential all study documentation, and take measures to prevent accidental or premature destruction of these documents. It is recommended that the Investigator retain the study documents for at least 5 years after the results from the trial have been reported to the regulatory authorities. However, applicable regulatory requirements should be taken into account in the event that a longer period is required. The Investigator must notify the Sponsor or it's representative prior to destroying any study essential documents within the five years period following the trial completion or discontinuation. The Investigator is responsible for ensuring that archiving can be maintained for five years locally, if this situation changes and archiving can no longer be ensured, the Investigator shall inform the Sponsor and the relevant records shall be transferred to a mutually agreed upon location.

14.7 Quality Control and Assurance

The quality control and assurance will be according to Royal Brompton & Harefield NHS Foundation Trust policies

14.8 Audits and Inspections

For the purpose of ensuring compliance with the Clinical Trial Protocol, Good Clinical Practice and applicable regulatory requirements, the Investigator should permit auditing by or on the behalf of the Sponsor and inspection by applicable regulatory authorities. The Investigator agrees to allow the auditors/inspectors to have direct access to his/her study records for review, it being understood that this person(s) are bound by professional confidentiality, and as such will not disclose any personal identity or personal medical information. The Investigator will make every effort to help with the performance of the audits and inspections, giving access to all necessary facilities, data and documents. As soon as the Investigator is notified of a future inspection by the authorities, he will inform the Sponsor and authorise the Sponsor to participate in this inspection. The confidentiality of the data verified and the protection of the patients should be respected during these inspections. The Sponsor or its representative will immediately communicate any results and information arising from the inspections by the regulatory authorities. The Investigator shall take appropriate measures required by the Sponsor to take correctives actions for all problems found during the audit or inspections.

14.9 Monitoring

14.9.1 Initiation Visit

Before the study commences each trial site will receive a training visit from CTEU where required. The purpose of these visits will be to ensure that the local research team (local principal investigator, co-investigators, study co-ordinator and pharmacists) fully understand the protocol, CRF and the practical procedures for the study.

14.9.2 Interim monitoring visits

At regular intervals during the study CTEU will perform monitoring visits to each trial site. The purpose of these visits is to ensure compliance with the protocol and that ethical and regulatory requirements are met. Source data verification (SDV) and checking of essential documents will be performed. Monitors will also visit the pharmacy departments to review study procedures, storage and accountability of IMP.

Monitoring visits also provide an opportunity for further training if required (e.g. new staff). Central review of study data will also be performed throughout the study by the data management team at CTEU.

14.9.3 Closeout Visit

At the end of the study each trial site will receive a closeout visit from CTEU to resolve any outstanding edit queries or adverse events and to verify the archiving procedures for study documentation.

15 PHARMACOVIGILANCE

15.1 Safety Reporting

The Sponsor has delegated responsibility for pharmacovigilance to the Clinical Trials and Evaluation Unit (CTEU) of the Royal Brompton & Harefield NHS Foundation Trust. The CTEU will be responsible for recording all reported serious adverse events from investigational trial sites, and expedited reporting of suspected

unexpected serious adverse reactions (SUSARs) in accordance with statutory regulations. Additional pharmacovigilance guidance will be provided in accompanying documents including CRFs, the Investigator Site File.

15.2 Definitions of AEs/ARs/SAEs/SUSARS

15.2.1 Definition Adverse Event

An adverse event (AE) is defined as any untoward medical occurrence in a patient or clinical trial subject administered a medicinal product and which does not necessarily have a causal relationship with this treatment.

An AE can therefore be any unfavourable and unintended sign (including an abnormal laboratory finding), symptom, or disease temporally associated with the use of an investigational medicinal product (IMP), whether or not considered related to the IMP.

15.2.2 Definition Adverse Reactions (AR)

An adverse reaction is defined as all untoward and unintended responses to an IMP related to any dose administered.

All AEs judged by either the reporting investigator or the sponsor as having reasonable causal relationship to a medicinal product qualify as adverse reactions. In the event an AR is reported during the trial, investigators will assess the severity of the adverse event using the following criteria, detailed on the adverse event report form in the case report form (CRF):

**Mild**:	Awareness of signs or symptoms, but easily tolerated; are of minor irritant type; causing no loss of time from normal activities; symptoms would not require medication.
**Moderate**:	Discomfort severe enough to cause interference with usual activities.
**Severe**:	Inability to do work or usual activities; signs and symptoms may be of systemic nature or require medical evaluation and/or treatment.

15.2.3 Definition Serious Adverse Events/Reactions

Serious adverse events or reactions can be defined as any untoward medical occurrence or effect that at any dose results in

- *death*
- *is life threatening*
- *requires hospitalisation or prolongation of existing inpatients hospitalisation*
- *results in persistent or significant disability or incapacity or is a congenital anomaly or birth defect.*

Life-threatening in the definition of a serious adverse event or serious adverse reaction refers to an event in which the subject was at risk of death at the time of event; it does not refer to an event which hypothetically might have caused death if it were more severe.

Medical judgement should be exercised in deciding whether an adverse event/reaction is serious in other situations. Important adverse events/ reactions that

are not immediately life-threatening or do not result in death or hospitalisation but may jeopardise the subject or may require intervention to prevent one of the other outcomes listed in the definition above, should also be considered serious.

In the event of pregnancy, follow-up of the pregnancy will be mandatory until the outcome has been determined. Pregnancy will be recorded as an AE in all cases. It will qualify as an SAE only if it fulfils SAE criteria.

15.3 Expectedness of AEs

An adverse reaction, the nature, or severity of which is not consistent with the applicable product information (summary of product characteristics (SmPC).

When the outcome of the adverse reaction is not consistent with the applicable product information this adverse reaction should be considered as unexpected. Side effects documented in the SmPC which occur in a more severe form than anticipated are also considered to be unexpected.

All expected drug related adverse reactions are detailed in the Summary of Product Characteristics (SmPC) for Prasugrel and Clopidogrel (Plavix).

15.4 Suspected Unexpected Serious Adverse Reactions (SUSARS)

A SAR can be considered unexpected when the adverse reaction, the nature or severity of which is not consistent with the applicable product information or expected serious adverse events listed above. All suspected unexpected serious adverse reactions (SUSARs) related to an IMP, which occur during the trial, are subject to expedited reporting.

15.5 Expected Serious Adverse Events (as a result of the underlying disease or study treatments):

In the context of this study the following SAEs would be considered possible as a result of ACS (including routine management and PCI) and the treatments under investigation:

1. Death
2. Myocardial Infarction
3. Recurrent angina
4. Stroke
5. Bleeding
6. Arrhythmias or heart block
7. Cardiac rupture or perforation
8. Tamponade
9. Rash
10. Valve damage or regurgitation
11. Embolic complications
12. Gastric upset

The reporting investigator reporting the event will make a judgement about expectedness. These events will be adjudicated by the Chief Investigator/or designated deputy.

15.6 Reporting of Serious Adverse Events/Reactions

In the event of an SAE, investigators will report details on the SAE form on the CRF, including date of event, admissions, diagnosis details, date of discharge or death. SAE reports must be completed and sent to CTEU within 24 hours of the investigator's knowledge of the SAE. Investigators may be required to submit reports should further information become available. Investigators are required to assess and assign causality and expectedness of each event on the form. The CTEU will review all SAE reports for completeness. SAEs will be adjudicated by the Chief Investigator/deputy who will review the SAE reports and inform CTEU of the assessment.

15.7 Expedited Reporting of SUSARs

- All SUSARs will be reported to the required Regulatory Authority and REC(s), by CTEU.
- This is an open label study so all SUSAR reports will be unblinded prior to submission.
- A SUSAR, which is fatal, or life threatening will be reported as soon as possible and within 7 days of knowledge of the event.
- A SUSAR, which is not fatal, or life threatening will be reported within 15 days of knowledge of the event.
- The CTEU will inform all relevant parties of any reported SUSARs within 15 working days.

15.8 Annual Reporting

The CTEU will submit annual safety reports of all serious adverse events / reactions including SUSARs reported during that period, in accordance with regulatory requirements to the regulatory authority and required REC(s). Annual safety reports will be submitted to the regulatory authority on the anniversary of the study's original clinical trials authorisation. Annual progress reports will be submitted to the appropriate REC.

16 TRIAL ORGANISATION AND COMMITTEES

16.1 Study Management

The study sponsor is The Royal Brompton & Harefield NHS Foundation Trust. The Chief Investigator will be Dr Miles Dalby at the Royal Brompton Hospital. Dr Tobias Geisler at University Hospital Tübingen has research experience with platelet function and will be the PI for Germany. A Trial Steering Committee and Data Monitoring Committee, and Trial Management Group will be convened to oversee the trial. Central co-ordination of this clinical trial will be provided by the Clinical Trials and Evaluation Unit (CTEU) under Dr Marcus Flather.

16.2 Trial Steering Committee (TSC)

The main role of the TSC is to monitor and supervise the progress of the trial. The composition of the Trial Steering Committee will comply with applicable guidelines with an independent Chair and lay representation as well as the Chief Investigator and main co-investigators. The TSC will meet regularly throughout the study.

16.3 Data Monitoring Committee (DMC)

All members of the DMC are independent of the trial. The DMC will meet prior to the start of the trial and then one and two thirds of the way through the Trial or as required thereafter. The DMC will be expected to develop, in agreement with the investigators, a charter outlining their responsibilities and operational details.

16.4 Study Co-ordination

The study will be co-ordinated and managed by the Clinical Trials and Evaluation Unit (CTEU) a dedicated clinical trials department within the Royal Brompton Hospital. In addition to providing overall project co-ordination, the CTEU will assist in preparing the final protocol, the investigators' manuals, design the Case Report Forms (CRF), provide the randomisation service and design and instigate the data management system. The CTEU will ensure that the trial runs according to the pre-agreed timetable, recruitment targets are met, CRFs are completed accurately, compliance with relevant ethical and regulatory standards, and that all aspects of the study are performed to the highest quality. The CTEU will also assist in the training of investigators and co-ordinators at the start-up of the study and for performing monitoring and pharmacovigilance procedures throughout.

16.5 Trial Sites

There are three participating sites:

Harefield Hospital UK
Freeman Hospital, UK
University Hospital Tübingen, Germany

Support for patient identification, study administration, data collection and follow-up will be provided to each participating Trial Site(s).

16.6 Investigators' Responsibilities

Investigators must ensure that local Institutional approval has been obtained as well as Agreements signed off by their Institution prior to the start of the study. Investigators are responsible for performing the study in accordance with the European Clinical Trials Directive, all local laws and guidelines. Investigators are required to ensure compliance to the Clinical Trial Protocol, CRFs, Investigators File and any other study instructions as required by the Sponsor or it's representatives. Investigators are required to ensure the accuracy of the trial data according to the instructions provided Investigators are required to allow access to study documentation or source data on request for monitoring visits and audits performed by the CTEU, the Sponsor or any regulatory authorities. The Investigator may appoint co-investigators to assist with conduct of the study locally. All co-investigators must be listed as members of the research team and appropriately trained. The Investigator has overall responsibility for ensuring the conduct of the study locally.

17 END OF TRIAL

17.1 Planned Termination

The trial will end when the last patient randomised has completed 30 days of follow up.

17.2 Premature Termination by Sponsor

The trial may be terminated prematurely:
- If the recruitment target cannot be met within the projected recruitment phase
- If the Trial Steering Committee believe the trial is no longer clinically relevant
- If there are significant concerns regarding the benefit / risk to the patient is in doubt.

The trial can be terminated by the Sponsor or it's representative at an individual Trial Site(s) if:

- the Trial Site(s) cannot comply with the requirements of the protocol,
- the Trial Site(s) is unable to comply with the required data standards,
- the required recruitment rate is not met.

If (after discussion between the Steering Committee, the Sponsors and the CTEU) the trial is terminated in a Trial Site(s) because of repeated serious protocol violations, or gross violations of data standards, all patients entered in the Trial Site(s) concerned will be excluded from analysis.

17.3 Premature Termination by the Investigator

The Investigator must provide prior notice to the Sponsor or it representative of his/her decision and give the reason in writing. In all cases the appropriate Ethics Committee and appropriate regulatory authority should be informed.

18 PUBLICATION POLICY AND DISSEMINATION OF RESULTS

The results from the trial will be submitted for publication in a major journal irrespective of the outcome. The Trial Steering Committee will be responsible for approval of all manuscripts arising from the study prior to submission for publication. Sub-studies of centre-specific data may only be carried out with the knowledge and approval of the Trial Steering Committee. Sub study publications must not be published prior to the publication of the main study.

All publications and presentations will make appropriate acknowledgement of the contribution of the collaborative group. At the end of the study, patients will be able to request a copy of the results of the study from the investigator at that site.

19 REFERENCES

[1] Sabatine MS, Hamdalla HN, Mehta SR, Fox KA, Topol EJ, Steinhubl SR, Cannon CP. Efficacy and safety of clopidogrel pretreatment before percutaneous coronary intervention with and without glycoprotein IIb/IIIa inhibitor use. Am Heart J. 2008;155(5):910-7.
[2] Geisler T, Grass D, Bigalke B, Stellos K, Drosch T; Dietz K, Herdeg C, Gawaz M. The PREDICT (Residual Platelet Aggregation after Deployment of Intracoronary Stent)-Score. J Thromb Haemostasis 2008;6(1):54-61.
[3] Geisler T, Anders N, Paterok M, Langer H, Stellos K, Lindemann S, Herdeg C, May AE, Gawaz M. Platelet response to clopidogrel is attenuated in diabetic patients undergoing coronary stent implantation. Diabetes Care. 2007;30(2):372-4.
[4] Geisler T, Schaeffeler E, Dippon J, Winter S, Buse V, Bischofs C, Zuern C, Moerike K, Gawaz M, Schwab M. Cytochrome P400 2C19 and non-genetic factors predict poor responsiveness to clopidogrel loading dose after coronary stent implantation. Pharmacogenomics 2008;9(9):1251-9.
[5] Mega JL, Close SL, Wiviott SD, et al. Cytochrome p-400 polymorphisms and response to clopidogrel. N Engl J Med 2009;360(4):354-62.
[6] Mega JL, Close SL, Wiviott SD, Shen L, Walker JR, Simon T, Antman EM, Braunwald E, Sabatine MS. Genetic variants in ABCB1 and CYP2C19 and cardiovascular outcomes after treatment with clopidogrel and prasugrel in the TRITON-TIMI 38 trial: a pharmacogenetic analysis. Lancet. 2010;376(9749):1312-9.
[7] Wallentin L, James S, Storey RF, Armstrong M, Barratt BJ, Horrow J, Husted S, Katus H, Steg PG, Shah SH, Becker RC; PLATO investigators..Effect of CYP2C19 and ABCB1 single nucleotide polymorphisms on outcomes of treatment with ticagrelor versus clopidogrel for acute coronary syndromes: a genetic substudy of the PLATO trial. Lancet. 2010 Oct 16;376(9749):1320-8.
[8] Geisler T, Langer H, Wydymus M, Göhring K, Zürn C, Bigalke B, Stellos K, May AE, Gawaz M. Low response to clopidogrel is associated with cardiovascular outcome after coronary stent implantation. Eur Heart J. 2006;27:2420-2425.
[9] Hochholzer W, Trenk D, Bestehorn HP, et al. Impact of the degree of peri-interventional platelet inhibition after loading with clopidogrel on early clinical outcome of elective coronary stent placement. J Am Coll Cardiol 2006;48(9):1742-50.
[10] Mehta SR, Tanguay JF, Eikelboom JW, Jolly SS, Joyner CD, Granger CB, Faxon DP, Rupprecht HJ, Budaj A, Avezum A, Widimsky P, Steg PG, Bassand JP, Montalescot G, Macaya C, Di Pasquale G, Niemela K, Ajani AE, White HD, Chrolavicius S, Gao P, Fox KA, Yusuf S; CURRENT-OASIS 7 trial investigators. Double-dose versus standard-dose clopidogrel and high-dose versus low-dose aspirin in individuals undergoing percutaneous coronary intervention for acute coronary syndromes (CURRENT-OASIS 7): a randomised factorial trial. Lancet. 2010;376(9748):1233-43.
[11] Montalescot G, Sideris G, Meuleman C, Bal-dit-Sollier C, Lellouche N, Steg PG, Slama M, Milleron O, Collet JP, Henry P, Beygui F, Drouet L. A randomised comparison of high clopidogrel loading doses in patients with non-ST-segment elevation acute coronary syndromes: the ALBION (Assessment of the Best Loading Dose of Clopidogrel to Blunt Platelet Activation, Inflammation and Ongoing Necrosis) trial. J Am Coll Cardiol. 2006;48:931–938.
[12] L'Allier PL, Ducrocq G, Pranno N, Noble S, Ibrahim R, Grégoire JC, Azzari F, Nozza A, Berry C, Doucet S, Labarthe B, Théroux P, Tardif JC, for the PREPAIR Study Investigators. Clopidogrel 600-mg double loading dose achieves stronger platelet inhibition than conventional regimens: results from the PREPAIR randomised study. J Am Coll Cardiol. 2008;51:1066 –1072.
[13] Bonello L, Dignat-George F, Camoin-Jau L, Barragan P, Arques S, Paganelli F. Adjusted clopidogrel loading doses according to vasodilator stimulated phosphoprotein phosphorylation index decrease rate of major adverse cardiovascular events in patients with clopidogrel resistance: a multicenter randomised prospective study. J Am Coll Cardiol. 2008;51:1404–1411.
[14] Di Sciascio G, Patti G, Pasceri V, Colonna G, Mangiacapra F, Montinaro A; on behalf of the ARMYDA-4 RELOAD investigators.Clopidogrel reloading in patients undergoing percutaneous coronary intervention on chronic clopidogrel therapy: results of the ARMYDA-4 RELOAD (Antiplatelet therapy for Reduction of MYocardial Damage during Angioplasty) randomised trial. Eur Heart J. 2010 Apr 2. [Epub ahead of print]
[15] Collet JP, Silvain J, Landivier A, Tanguy ML, Cayla G, Bellemain A, Vignolles N, Gallier S, Beygui F, Pena A, Montalescot G. Dose effect of clopidogrel reloading in patients already on 75-mg

maintenance dose: the Reload with Clopidogrel Before Coronary Angioplasty in Subjects Treated Long Term with Dual Antiplatelet Therapy (RELOAD) study. Circulation. 2008;118(12):1225-33.

[16] Bonello L, Camoin-Jau L, Armero S, Com O, Arques S, Burignat-Bonello C, Giacomoni MP, Bonello R, Collet F, Rossi P, Barragan P, Dignat-George F, Paganelli F. Tailored clopidogrel loading dose according to platelet reactivity monitoring to prevent acute and subacute stent thrombosis. Am J Cardiol. 2009;103(1):5-10.

[17] Hagihara K, Kazui M, Kurihara A, Yoshiike M, Honda K, Okazaki O, Farid NA, Ikeda T. A possible mechanism of the differences in efficiency and variability of active metabolite formation from thienopyridine antiplatelet agents, prasugrel and clopidogrel. Drug Metab Dispos. 2009; 37(11):2145-52

[18] Rehmel JL, Eckstein JA, Farid NA, Heim JB, Kasper SC, Kurihara A, Wrighton SA, Ring BJ. Interactions of two major metabolites of prasugrel, a thienopyridine antiplatelet agent, with the cytochromes P400. Drug Metab Dispos. 2006; 34(4):600-7.

[19] Wallentin L, Varenhorst C, James S, Erlinge D, Braun OO, Jakubowski JA, Sugidachi A, Winters KJ, Siegbahn A. Prasugrel achieves greater and faster P2Y12 receptor-mediated platelet inhibition than clopidogrel due to more efficient generation of its active metabolite in aspirin-treated patients with coronary artery disease. Eur Heart J. 2008; 29(1):21-30.

[20] Wiviott SD, Braunwald E, McCabe CH, Montalescot G, Ruzyllo W, Gottlieb S, Neumann FJ, Ardissino D, De Servi S, Murphy SA, Riesmeyer J, Weerakkody G, Gibson CM, Antman EM; TRITON-TIMI 38 Investigators. Prasugrel versus clopidogrel in patients with acute coronary syndromes. N Engl J Med. 2007; 357: 2001-2015.

[21] Unger EF. Weighing benefits and risks--the FDA's review of prasugrel. N Engl J Med. 2009; 361(10):942-5.

[22] National Institute for Health and Clinical Excellence. Final appraisal determination – Prasugrel for the treatment of acute coronary syndromes with percutaneous coronary intervention. Issue date: August 2009.

[23] Wallentin L, Becker RC, Budaj A, Cannon CP, Emanuelsson H, Held C, Horrow J, Husted S, James S, Katus H, Mahaffey KW, Scirica BM, Skene A, Steg PG, Storey RF, Harrington RA; PLATO Investigators, Freij A, Thorsén M. Ticagrelor versus clopidogrel in patients with acute coronary syndromes. N Engl J Med. 2009;361(11):1045-57.

[24] Sibbing D, Braun S, Morath T, Mehilli J, Vogt W, Schömig A, Kastrati A, von Beckerath N. Platelet reactivity after clopidogrel treatment assessed with point-of-care analysis and early drug-eluting stent thrombosis. J Am Coll Cardiol. 2009 Mar 10;53(10):849-56.

[25] Müller-Schunk S, Linn J, Peters N, Spannagl M, Deisenberg M, Brückmann H, Mayer TE. Monitoring of clopidogrel-related platelet inhibition: correlation of nonresponse with clinical outcome in supra-aortic stenting. AJNR Am J Neuroradiol. 2008 Apr;29(4):786-91.

[26] Sibbing D, Braun S, Jawansky S, Vogt W, Mehilli J, Schömig A, Kastrati A, von Beckerath N. Assessment of ADP-induced platelet aggregation with light transmission aggregometry and multiple electrode platelet aggregometry before and after clopidogrel treatment. Thromb Haemost. 2008;99(1):121-6.

APPENDIX A: DESCRIPTION OF PLATELET FUNCTION ANALYISIS (MULTI-PLATE®)

Multi-Plate ® analysis is based on impedance aggregometry which. This methods utilizes the fact that blood platelets are non-thrombogenic in their resting state, but expose receptors on their surface when activated which allow them to attach on vascular injury and artificial surfaces. In each test cell there are two parallel pairs of electrodes. Activate platelets have a tendency to adhere and aggregate on the metal sensor wires - made of highly conductive copper which is silver coated. Platelet adhesion onto the sensor wires results in a rise in the electrical resistance which is continuouslyregistered for a period of six minutes(See Figure 1). Platelet aggregation will be measured followed by agonist stimulation using adenosine diphosphate (ADP) in this study. Measurement of ADP-induced platelet aggregation has been documented sufficient sensitivity to monitor platelet inhibitory effects of ADP-receptor blockers.

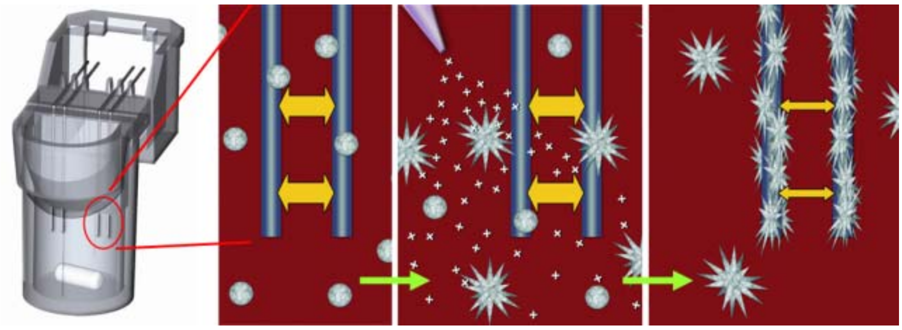

Figure 1: Diagram of the Multi-Plate ® measurement system.

SOP- reagents and protocol for automated measurement

Reagent: **ADPtest**

Stability: Reconstituted and refrigerated at 2-8 ° C: 7 days

Reconstituted and frozen at -20 ° C: 4 weeks (17 h stable after thawing)

Unresconstituted at 2-8 ° C: see expiration date on label reagents

Reconstitution: Dissolve with 1.0 ml of distilled water. Swirl gently before use, do not shake and let stand for 10 min at RT. The reagent should be colourless and clear.

Reagent: **Prostaglandin E1 (for ADPT HS (high sensitivity))**

Dissolve with 1.0 ml of distilled water. Swirl gently before use, do not shake and let stand for 10 min at RT. The reagent should be colourless and clear.

Stability: Reconstituted and refrigerated at 2-8 ° C: 7 days

Reconstituted and frozen at -20 ° C: 4 weeks (17 h stable after thawing)

Unresconstituted at 2-8 ° C: see expiration date on label reagents

Automated measurement Multi-Plate

Click F1: Auto pipette.
- Enter the patient ID and select test. Maximum 5 tests can be created and run in parallel.
- Confirm “start”
- Place flow cells in to the appropriate channels.
- Connect the sensor cable to the measuring cells.
- Confirm with the "Next" - Button.
- Perform the pipette sequence as displayed, by pressing the blue Start button (see Figure 2) on the electronic pipette.

Figure 2: Multi-Plate ® system electronic pipette.

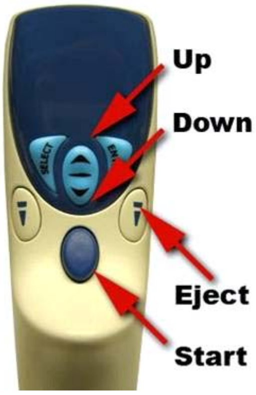

- The current pipette step is highlighted with a green arrow. Executed steps are checked off.
- When using Hirudinised or Li-Heparin anticoagulated blood all measurement cells are filled with 300 µl saline and 300 µl sample. Citrated blood requires 300 µl and 300 µl NaCl/CaCl2
- Then follows a 3-minute incubation at 37 ° C (automatically).
- After the incubation period a tone sounds and the activator (ADP) must be added.
- After adding the activator to the respective channel, measurement starts automatically and in the channel window displays the icon:

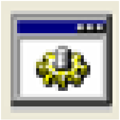

- After 6 minutes the test is completed and the channel window diplays the icon

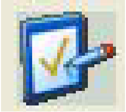

- Print out the results of the selected channels by selecting the function button "F6: Print".
- After the measurement remove the sensor cables from the flow cells. Also dispose of the measurement cells from the measurement positions. Then clear the channels via the function button "F7: Clear Channel “.

The analysis is specified by the AUC (Area Under the Curve = area under the aggregation curve) in U (units).
