# Supplementary material for: High Platelet Reactivity in Patients with Acute Coronary Syndromes Undergoing Percutaneous Coronary Intervention: Randomised Controlled Trial Comparing Prasugrel and Clopidogrel
Source: PLoS One. 2015 Aug 28;10(8):e0135037. doi: 10.1371/journal.pone.0135037 (PMC4552627; doi:10.1371/journal.pone.0135037)
Supplement: S1 Table — (DOCX) [file pone.0135037.s003.docx]

S1 Table: Reasons for no enrolment

| **Reason** | **N** |
| --- | --- |
| Other antiplatelet therapy (including GPIIb/IIIa inhibitors) besides aspirin | 208 |
| Patients <18 years and ≥75 years | 179 |
| Missed | 38 |
| Declined | 35 |
| Unknown | 34 |
| IOM | 32 |
| No PCI | 32 |
| History of stroke or transient ischemic attack | 27 |
| Body weight <60kg | 19 |
| Cath lab time | 16 |
| Co-morbidities | 13 |
| Insufficient time to consent | 12 |
| Gastrointestinal bleeding or active peptic ulceration | 9 |
| Pre-treatment with prasugrel within 7 days of randomisation | 8 |
| Needs coronary artery bypass surgery | 8 |
| Oral anticoagulation | 7 |
| Compliance | 7 |
| Enrolled other research project | 7 |
| Not randomised PA < 40 AUC | 5 |
| Location | 5 |
| Not on clopidogrel | 5 |
| Patients with increased bleeding risk. | 4 |
| Intracranial neoplasm, arteriovenous malformation or aneurysm. | 4 |
| No stent | 4 |
| Recent major trauma or surgery | 3 |
| Non-steroidal anti-inflammatory drugs (NSAIDs) or cyclooxygenase-2 (COX-2) inhibitors | 3 |
| Too anxious | 3 |
| Internationally Normalized Ratio (INR)> 1.5 at the time of screening | 2 |
| Language | 2 |
| Elective PCI | 2 |
| Tx and return | 2 |
| Hb<10g/dL | 1 |
| Severe hepatic impairment (Child Pugh class C) | 1 |
| Female patients -pregnant/planning pregnancy/not using reliable contraception/ breastfeeding | 1 |
| PPCI | 1 |
| Reloaded with clopidogrel | 1 |
| PCI > 72 hours from admission | 1 |
